# Identification and Characterization of the Sucrose Synthase 2 Gene (*Sus2*) in Durum Wheat

**DOI:** 10.3389/fpls.2016.00266

**Published:** 2016-03-10

**Authors:** Mariateresa Volpicella, Immacolata Fanizza, Claudia Leoni, Agata Gadaleta, Domenica Nigro, Bruno Gattulli, Giacomo Mangini, Antonio Blanco, Luigi R. Ceci

**Affiliations:** ^1^Department of Biosciences, Biotechnologies and Biopharmaceutics, University of Bari “A. Moro”Bari, Italy; ^2^Department of Agricultural and Environmental Sciences, University of Bari “A. Moro”Bari, Italy; ^3^Department of Soil, Plant and Food Sciences Section Genetics and Plant Breeding, University of Bari “A. Moro”Bari, Italy; ^4^Institute of Biomembranes and Bioenergetics – National Research CouncilBari, Italy

**Keywords:** durum wheat, sucrose synthase, thousand kernel weight, starch, grain

## Abstract

Sucrose transport is the central system for the allocation of carbon resources in vascular plants. Sucrose synthase (SUS), which reversibly catalyzes sucrose synthesis and cleavage, represents a key enzyme in the control of the flow of carbon into starch biosynthesis. In the present study the genomic identification and characterization of the *Sus2-2A* and *Sus2-2B* genes coding for SUS in durum wheat (cultivars Ciccio and Svevo) is reported. The genes were analyzed for their expression in different tissues and at different seed maturation stages, in four tetraploid wheat genotypes (Svevo, Ciccio, Primadur, and 5-BIL42). The activity of the encoded proteins was evaluated by specific activity assays on endosperm extracts and their structure established by modeling approaches. The combined results of sucrose synthase 2 expression and activity levels were then considered in the light of their possible involvement in starch yield.

## Introduction

Starch is the major reserve carbohydrate in plants and its production is critical to both yield and overall quality of grain. It constitutes approximately 70% of mature grain dry weight and its characteristics directly affect on the nature and quality of flour and related products (Kim et al., 2003). Sucrose Synthase (SUS) catalyzes the reversible reaction of sucrose hydrolysis into UDP-glucose and fructose, the first step in the conversion of sucrose to starch (Baroja-Fernández et al., 2003; Keeling and Myers, 2010).

In the plant species examined to date, SUS isoforms are encoded by a small gene family. In *Arabidopsis thaliana* (Bieniawska et al., 2007) and rice (Hirose et al., 2008) there are at least six *Sus* genes, seven have been reported in *Populus tomentosa* (Zhang et al., 2010). In maize (*Zea mays*) the paralogous genes *shrunken1* and *Sus1* encode two biochemically similar isozymes, SUS1 and SUS2 (Chourey et al., 1998). The first one is the structural gene for the major endosperm isoform of the enzyme that is anaerobically induced in roots and shoots, while the second is the structural gene for the constitutively expressed isoenzyme in embryo and other tissues. Similar genes corresponding to the SUS1 and SUS2 proteins are also present in barley (Sánchez de la Hoz et al., 1992) and in wheat (Martinez de Ilarduya et al., 1993). In wheat, *Sus2* gene is predominantly expressed in the endosperm, while *Sus1* gene is also expressed in roots and leaves, where it is induced under conditions of anaerobiosis and low temperature (Maraña et al., 1990). Specifically in wheat, *Sus1* and *Sus2* genes are located on homoeologous group 7 chromosomes and on homoeologous group 2 chromosomes, respectively (Jiang et al., 2011; Hou et al., 2014).

Up to now, the reasons for the presence of SUS isoforms have not been clarified. Different isoforms may carry out identical functions within the cell but operate in distinct cell types, developmental periods, or environmental conditions. However, it is also possible that different isoforms may have distinct, non-overlapping functions within the same cell. For example, mutations in *Shrunken1* and *Sus1* genes in maize (Chourey et al., 1998) eliminate isoforms of SUS that are highly expressed in the developing seed, resulting in reductions in seed SUS activity and reduced accumulation of starch. Furthermore, tubers of transgenic potato plants with reduced SUS activity also accumulate less starch than wild-type tubers (Zrenner et al., 1995).

The biophysical analyses on SUS protein from barley (Guerin and Carbonero, 1997), maize (Hardin et al., 2004; Duncan et al., 2006), mung bean (Nakai et al., 1998), and potato (Römer et al., 2004), strongly suggested that the native enzyme is a tetramer, although SUS can exist in a dimer form (Duncan and Huber, 2007). The crystal structure of SUS1 from *A. thaliana* (AtSus1) has been determined both as complex with UDP-glucose and as complex with UDP and fructose, at 2.8 and 2.85 A° resolutions, respectively (Zheng et al., 2011). The typical SUS sequence structure shows an N-terminal domain involved in cellular compartimentalization (residue 1–276) (Hardin et al., 2006); a GT-B glycosyltransferase (residues 277–776); and finally a C-terminal extension of 32 residues, which is the most variable among the SUS domains (Barrero-Sicilia et al., 2011).

In 89 modern wheat varieties of a Chinese mini-core collection, three single nucleotide polymorphisms (SNPs) in *TaSus2-2B* form two haplotypes, Hap-L and Hap-H (Jiang et al., 2011). The analysis was further extended to a total of 1520 wheat accessions, including 348 modern Chinese cultivars (Hou et al., 2014). The association analysis suggested a strong relationship between the two haplotypes and an important agricultural trait, known as thousand grain weight (TGW). To date, few genome sequencing data have been released in common wheat^1^, and no information is available for *Sus* genes sequences nor their relation with yield components in durum wheat. Thus, the aim of this study is the genomic identification and characterization of *Sus2-2A* and *Sus2-2B* genes in the cultivars Ciccio and Svevo of durum wheat *Triticum turgidum* L. sp. *durum* (Desf.) Husnot, chosen for their differences in important qualitative and quantitative traits, including grain yield components (Blanco et al., 2012; Pasqualone et al., 2015). The genes were analyzed for their expression levels in different tissues and at different maturation stage of seed, and the results obtained were compared to other two wheat genotypes (Primadur and 5-BIL42). The activity of the encoded proteins was evaluated by specific activity assays on endosperm extracts and their structure established by modeling approaches.

## Materials and Methods

### Plant Material

Three durum wheat cvs Ciccio, Svevo and Primadur, and one durum wheat breeding line, 5-BIL42, were used for DNA and RNA preparation and for SUS2 activity assays. Svevo and Primadur are cvs with low thousand kernel weight (TKW), while Ciccio and 5-BIL42 have higher values (Blanco et al., 2012; Pasqualone et al., 2015). Moreover, cvs Ciccio and Svevo are parental lines of a mapping population represented by a set of 120 recombinant inbred lines (RILs) as previously described (Gadaleta et al., 2009). Plants were grown in field trial at Valenzano (Bari, Italy) and leaves and spikes were collected according to specific experimental procedures at different phenological stages (first leaf, medium milk stage, and hard dough stage).

A complete set of nulli-tetrasomic lines (NTs) developed by Sears (1966) from the common wheat (*Triticum aestivum* L. sp. *aestivum*) cv Chinese Spring were used to physically localize Sus2 markers to specific chromosomes.

### *TaSus2* Gene Sequence Determination for Chinese Spring Cultivar

In order to identify the complete genomic sequences of the three *TaSus2* genes in hexaploid wheat, an available Chinese Spring genomic sequence in the cereals database^2^ was used. The cDNA sequence of *TaSus2* gene (GenBank accession No. AJ000153.1) was used as query in a blast analysis to identify and to extract the raw sequencing data from the database. The 454 reads were assembled using the SeqMan software by DNAStar (Lasergene).

### Gene Identification in the Durum Wheat cvs Ciccio and Svevo

Genomic DNA was isolated from fresh leaves using the GeneElute Plant Genomic Miniprep Kit (Sigma).

PCR amplifications were carried out using 80 ng of genomic DNA, 5 μl 10X Buffer, 1 μl 10 mM dNTPs, 2 μl 10 μM forward and reverse primers, 2U of Triple-Master Polymerase Mix (Eppendorf). After the first DNA denaturation step at 94°C for 3 min, amplifications were run for 30 cycles consisting of 1 min at 94°C, 1 min at 69°C, and 1 min at 72°C. A final elongation step was then run at 72°C for 7 min. PCR products were analyzed on 1.5% agarose gel and purified with the PCR product purification kit “GFX^TM^ PCR DNA and Gel Band Purification” Kit (GE Healthcare). The purified amplifications were then cloned using the pGem-T Easy vector (Invitrogen) and transformed into JM109 competent *Escherichia coli* cells by the heat shock method. Positive clones were selected and their plasmids were sequenced at both insert ends with SP6 and T7 primers. The full fragment sequence was obtained by primer walking using specific primers (Supplementary Table S1). Regions of ambiguous reads were re-sequenced with additional primers.

### RNA Extraction

Leaf and spike samples of the cultivars were collected at different phenological stages from three biological replicates. Tissues from each sample were immediately frozen in liquid nitrogen and stored at –80°C until RNA extraction.

Total RNA was extracted from leaf samples using the PureLink RNA Micro kit (Invitrogen) according to manufacturer’s instructions. Differently, total RNA was isolated from caryopses using a phenol method. Hundred milligram of starting material (three or four grains) were homogenized in liquid nitrogen and resuspended in buffer A (20 mM Tris pH 9, 200 mM NaCl, 1% Sarcosyl, 20 mM EDTA, 5 mM DTT). RNA was first extracted using phenol/chloroform/isoamylic alcohol (IAA) (25:24:1), then the resulting aqueous phase was extracted by Trizol and chloroform/IAA (24:1) and finally the aqueous phase was extracted by chloroform/IAA (24:1). RNA was then precipitated with two volumes of ice cold 100% ethanol and 1/10 volume of sodium acetate, pH 5.2 at –80°C for 30 min. After centrifugation, the pellet was washed with 70% ethanol, air dried and then resuspended in the appropriate volume of DEPC-treated water.

The resulting RNA was treated with DNase–Rnase Free (PureLink^®^ DNase Invitrogen) directly on column, according to manufacturer’s instructions, and eluted in DEPC-treated water. The total amount of RNA and its purity were determined using Nano-Drop ND1000 spectrophotometer, and checked on 1.5% agarose gels. Absence of genomic DNA contamination was checked by specific amplifications (data not shown).

### RT-PCR and qRT-PCR Expression Analysis

#### RT-PCR

One microgram of RNA was used for ss-cDNA synthesis in the presence of oligodT primer. Ss-cDNA synthesis was carried out by using the SuperScript First-Strand Synthesis System (Life Technologies) according to manual instructions. PCR amplifications were performed using the following pairs of primers (Supplementary Table S1): Sus2-167for/ Rev1-ciccio, FOR-ESII/REV-ESVI, FOR-ESVI /REV-ESIX, FOR-ESX/REV-ESXII, FOR-ESXII/REV-ESXIII, FOR-ESXIII/REV-ESXV.

#### qRT-PCR

Diluted ss-cDNA (1:3) was used in all the qRT-PCR amplifications. Reactions were performed using the following pairs of primers: RT-9E-FOR A/RT-10E-REVA, RT-9E-FORB/RT-10E-REVB,CDC-FOR/CDC-REV, RLI-FOR/RLI-REV, ADP-RF-FOR/ADP-RF-REV (Supplementary Table S1). Genes coding for ADP-Ribosylation Factor (ADP-RF) and RNase L Inhibitor-like protein (RLI) were used as reference genes (Andersen et al., 2004; Paolacci et al., 2009; Giménez et al., 2011; Nigro et al., 2013) (Supplementary Table S1).

Data from qRT-PCR experiments for both *Sus2* and endogenous control genes are the mean values of three independent amplification reactions carried out on three different plants harvested at the same phenological stage. qRT-PCR experiments were performed on the Quant Studio 6 Flex Real-Time PCR System (Applied Biosystems, Life Technologies), using 1 μl of diluted cDNA as template for each reaction with SYBR Green PCR Master Mix (Life Technologies). No template controls were included as negative controls for each primer pair. Amplification parameters were as follows: hot start at 95°C for 15 min; 40 amplification cycles (94°C for 15 s, 60°C for 30 s); dissociation curve step (95°C for 15 s, 60°C for 15 s, 95°C for 15 s). The specificity of the amplicons was confirmed by the presence of a single band of expected size for each primer pair in agarose gels (2% w/v), by single peak melting curves of the PCR products, and by the sequencing of the amplified fragments (MWG Operon Eurofins, Germany). Fluorescence raw data were exported by the Flex Real Time PCR System Software (Applied Biosystems, Life Technologies) and analyzed using the DART-PCR Excel workbook (Peirson et al., 2003). Actual amplification efficiency values (*E*) for each amplicon were used to correct Cq values before analyzing these data by the ΔCq method to compare relative expression results. Expression levels were calculated as already described (Volpicella et al., 2014). All the results were analyzed by ANOVA.

### 3′-RACE (Rapid Amplification of 3′-cDNA Ends Analysis)

For 3′ RACE, single strand cDNA was synthesized starting from 1 μg of total RNA and 1 μl of 50 mM oligodT, using the SuperScript First-Strand Synthesis System (Life Technologies) according to manufacture’s instructions. The cDNA obtained was then used as substrate for a first PCR reaction using a gene-specific primer (GSP- FOR A-3RACE for genome A and GSP-FORB-3RACE for genome B) and an anchored-oligo dT primer that targets the poly(A) tail region. PCR products were then subjected to a nested PCR using a gene-specific primer (GSP1- FORA2-3RACE for genome A and GSP-FORB2-3RACE for genome B) and an anchored primer (Supplementary Table S1).

### Structural Modeling and Multiple Sequence Alignments

The three dimensional superimposition of SUS2 protein with the AtSus1 from *A. thaliana* was achieved using the Phyre2 software (Kelley and Sternberg, 2009) and then visualized using the PyMOL (molecular visualization software system) program (Ordog, 2008).

The SUS2 amino acid sequences from mono- and dicotiledon plants species were obtained from NCBI database, such as *T. aestivum*, CAA03935.1; Brachypodium *(Brachypodium distachyon*), XP_003562658.1; maize *(Zea mays*), NP_001105323.1; rice (*Oryza sativa* Japonica Group), ABL74568.1; Populus *(Populus tomentosa)*, ADW80587.1; grape *(Vitis vinifera)*, XP_002271896.1; *Arabidopsis (Arabidopsis thaliana)*, NP_199730.1, and selected sequences were compared by alignment using the Clustal omega program.

### SUS Activity Assay

Sucrose was indirectly determined by measuring the D-fructose produced upon hydrolysis, using a slightly modified version of the Seliwanoff test (Shahidullah and Khorasani, 1972) described by Jiang et al. (2004). Enzyme activity was measured on endosperm extracts of kernels at milk stage after dissection from pericarp and embryo. Hundred microgram of starting material (three or four grains) were weighed and embryos were selectively removed. Endosperm was then homogenized in ice with a pestle, in the presence of 2.5 ml of 50 mM HEPES-NaOH (pH 7.5), 10 mM MgCl_2_, 2 mM EDTA, 50 mM 2-mercaptoethanol, 12.5% (V/V) glycerol, and 5% (W/V) insoluble PVP (polyvinylpyrrolidone-40). The homogenate was centrifuged at 10000 × *g* at 4°C for 10 min and the supernatant solution was used to estimate the activity of SUS2 enzyme. Further purification was not performed in order to avoid enzyme activity loss. The background value of each sample was determined in a parallel experiment by adding the same volume of thermally denatured enzyme solution.

Sucrose synthase was assayed in the direction of sucrose formation in a reaction mixture of 875 μL containing 50 mM HEPES-NaOH (pH 7.5), 50 mM fructose, 50 mM UDP-glucose, 15 mM MgCl_2_. The reaction was started by adding 80 μL of enzyme crude extraction. After 30 min at 30°C, the reaction was stopped in boiling water for 1 min. Then 0.1 mL of 1 M NaBH_4_ in 2 M NaOH was added to convert residual D-fructose into a mixture of D-sorbitol and D-mannitol. The resulting solution was heated in boiling water for 10 min and then cooled to 0°C, after which 3.5 mL of 30% HCl and 1 mL of 0.1% resorcinol were added, and the solution heated in 80°C water for 10 min. The formation of UDP-glucose-dependent sucrose catalyzed by SUS2 was then monitored at 480 nm with a UV/Visible Ultraspec 3000 spectrophotometer (Pharmacia Biotech). Enzyme activities were expressed as nanomoles of sucrose product per min and per mg of kernels at 30°C.

## Results

### Assembly of the *Sus2* Gene Sequence from Common Wheat (cv Chinese Spring)

The cDNA sequence of *TaSus2* gene (AJ000153.1) was used to search for corresponding gene sequences among the Chinese Spring draft genome assembly of gene-rich regions^2^, thus retrieving 11 overlapping contigs. Their assembly allowed us to obtain a single sequence composed of 15 exons and 14 introns (not shown) to be used for the isolation of the durum wheat homoeologous genes.

### Isolation and Sequencing of *Sus2* Genes in Durum Wheat cvs Ciccio and Svevo

The genomic DNA of the durum wheat cvs Svevo and Ciccio were amplified with the primer pair Sus2-167for and Sus2-168rev (Jiang et al., 2011; Supplementary Table S1), and the resulting fragments were cloned and sequenced. Only two different DNA sequences of 4344 and 4347 nucleotides were found in each durum cultivar, which were aligned with *TaSus2-2A* and *TaSus2-2B* of Chinese Spring, allowing us to putatively identify their genome assignment. The identity of *Sus2-2A* sequences between Chinese spring and durum wheat was 99.43%, while it was 99.88% for the *Sus2-2B* sequences. No polymorphisms were found within A and B genome sequences between Svevo and Ciccio durum cultivars, while few INDELs and several SNPs were detected between the two homoeologous sequences (Supplementary Figure S1). Interestingly, a single SNP in the fifth exon allowed the creation of an *EcoRI* restriction site, polymorphic between homoeologous sequences.

To genetically map *Sus2* genes in durum wheat, two specific reverse primers SUS2-REV7 and SUS2-REV9 to be coupled with primer FOR-5PROM were designed on the assembled Chinese Spring *TaSus2* genomic sequence at 600 nucleotides upstream of the ATG starting codon (Supplementary Table S1 and Figure S2). The amplified fragments were cloned and 20 recombinant plasmids were sequenced. No polymorphism were found in the upstream region of Ciccio and Svevo *Sus2* genes, and therefore a genetical map of the *Sus2* genes was not possible.

### Chromosome Mapping of the *Sus2* Genes

To physically map the two *Sus2* genes and validate the putative genome assignment of the two DNA sequences obtained, a set of NTs for all chromosome groups was amplified with the same genomic-specific primer pairs (Supplementary Table S1 and Figure S2), leading to the same PCR products (Supplementary Figure S3). By taking advantage of the previously identified *EcoRI* restriction site, the amplified fragments were then digested with *EcoRI* restriction enzyme. Only the N2A-T2B NT showed a digestion pattern, allowing the physical mapping of the *Sus2-2A* and *Sus2-2B* homoeologous sequences (Supplementary Figure S3).

**FIGURE 1:**
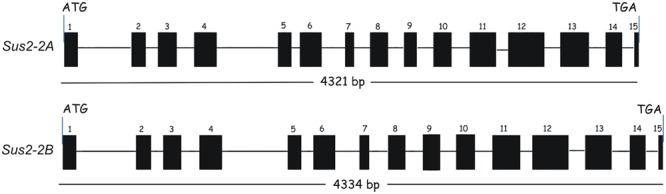
**Exon/Intron structure of durum wheat *Sus2* genes in Svevo and Ciccio durum wheat cultivars.** Exons are indicated by numbered boxes; introns are indicated by lines. Start and stop codon positions of the coding regions are marked.

From the ATG starting codon to the TAG stop codon the two genes show a similar length: the *Sus2-2A* gene is 4321 bp long, while the *Sus2-2B* is 4334 bp. Each gene is constituted of 15 exons and 14 introns (**Figure 1**). To validate the exon/intron organization of the two durum wheat *Sus2* genes, several combinations of primers were designed and used in RT-PCR experiments. The sequences obtained were identical to those resulting from the DNA genomic amplification, and no multiple cDNA fragments have been found. Six borders were found different from that hypothesized using the alignment against the cDNA sequence AJ000153.1 (data not shown). The intron border sequences do not always match the plant consensus intron borders (GT….AG) (Supplementary Figure S1).

The identical tetraploid Svevo and Ciccio DNA sequences are available at NCBI under accessions LN869542 for the 2A gene and LN869543 for the 2B gene.

### 3′RACE

The *Sus2* homoeologous genes were also compared for their 3′ untranslated region obtained by 3′ RACE in the two durum cvs Ciccio and Svevo. On the basis of genomic sequences, two pairs of forward primers corresponding to the *Sus2-2A* and *Sus2-2B* sequences (Supplementary Table S1 and Figure S3), to be used for the PCR and hemi-nested PCR steps of the 3-RACE, were designed.

3′ RACE was carried out on single-strand cDNA synthesized using RNA extracted from Ciccio and Svevo caryopses at medium milk stage starting from an oligo-dT primer. Double-stranded cDNA molecules were obtained by PCR using oligonucleotides FORA-3RACE or FORB-3RACE and the anchor primer. The PCR amplification products were then subjected to hemi-nested PCR, using the primers FORA2-3RACE or FORB2-3RACE as novel forward primers (Table S1). Alignments of sequences obtained from the RACE experiments are reported in Figure S1.

### Expression Profile of *Sus2* Genes in Different Durum Wheat Cultivars

**FIGURE 2:**
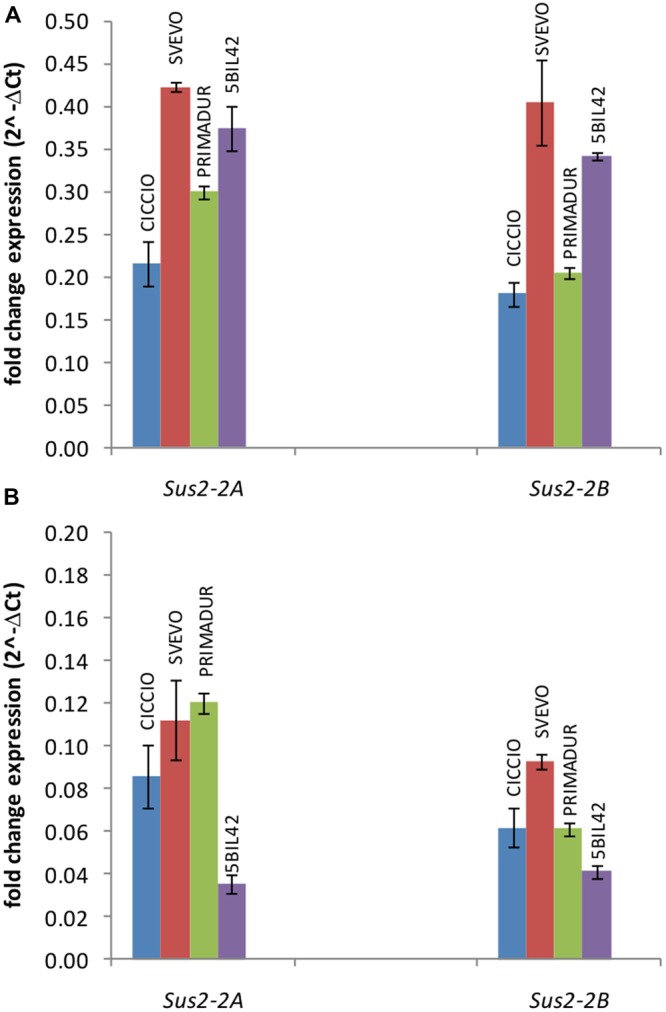
**qRT-PCR of *Sus2-2A* and *Sus2-2B* in the durum wheat cvs Ciccio, Svevo, Primadur, and 5-BIL42.** Comparison of the two homoeologous gene expression levels during the milk **(A)** and dough **(B)** stages. Error bars indicate the ±SD of mean values.

The expression level of *Sus2-2A* and *Sus2-2B* genes was estimated in kernels at different phenotypic stages (flowering stage, medium milk stage, and hard dough stage) and leaves of the wheat cvs Svevo, Ciccio, and Primadur, and of the durum wheat breeding line 5-BIL42. Total RNA was extracted from plants grown in field conditions and reverse-transcribed for qRT-PCR analyses. qRT-PCR reactions were performed using specific primer pairs designed in regions of the ninth and the tenth exons containing three SNPs (Supplementary Table S1 and Figure S2), to preferentially amplify the A and B genomic DNA sequences. No expression was observed in kernels at flowering stage and leaves, as already reported for the *T. aestivum* genes (Jiang et al., 2011). For all the genotypes analyzed the expression level of allelic genes in kernels at milk stage was significantly higher (*P* < 0.001) than in kernels at dough stage (**Figure 2**). In particular, the *Sus2-2A* and *Sus2-2B* relative transcript abundances in kernels at milk stage were 0.22 and 0.18 in cv Ciccio, 0.42 and 0.41 in cv Svevo, 0.30 and 0.21 in cv Primadur, 0.37 and 0.34 in cv 5-BIL42. At dough stage, transcript levels were 0.09 and 0.06 in cv Ciccio, 0.11 and 0.09 in cv Svevo, 0.12 and 0.06 in cv Primadur, 0.03 and 0.04 in breeding line 5-BIL42, for A and B homoeologous genes, respectively. Analysis of variance did not show significant differences between biological and technical replicates, thus demonstrating a stability of gene expression even in field conditions.

### SUS Activity

**FIGURE 3:**
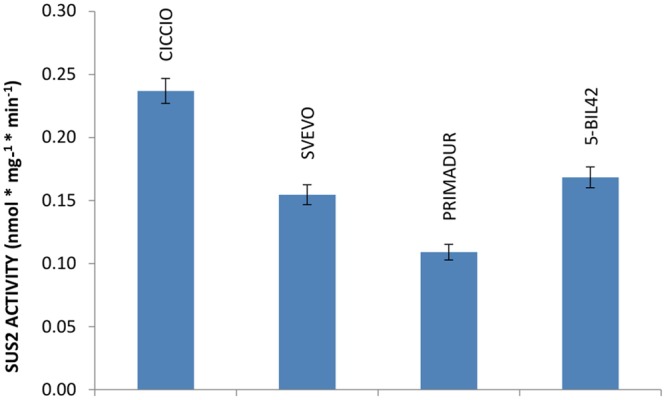
**Level of SUS2 activity in the durum wheat cvs Ciccio, Svevo, Primadur, 5-BIL42.** Sucrose hydrolytic activity in endosperm extracts is reported. Error bars indicate the ±SD of mean values.

The activity of SUS2 protein in cvs Ciccio, Svevo, Primadur, and in breeding line 5-BIL42 was estimated by measuring the amount of sucrose produced by the enzyme in the endosperm of kernels at milk stage, using UDP-glucose and D-fructose as substrates. Differently from other reports, we could verify that treatment with a strongly basic solution was not suitable for a satisfactory removal of residual D-fructose (Jiang et al., 2004). Conversely, we found that unreacted fructose can be conveniently removed by treatment with a basic NaBH_4_ solution. Quantification of sucrose was based on calibration curves obtained with sucrose solutions of known concentration. Specifically, the level of SUS2 activity measured as nmol^∗^mg^-1∗^min^-1^ was 0.24 in cv. Ciccio, 0.15 in cv. Svevo, 0.11 in cv. Primadur, and 0.17 in breeding line 5-BIL42 (**Figure 3**). Data from enzyme activity represent the mean values of three independent reactions carried out on three different plants harvested at the same phenological stage.

### Comparison of Amino Acid Sequences from Other Species and Three-Dimensional Modeling

**FIGURE 4:**
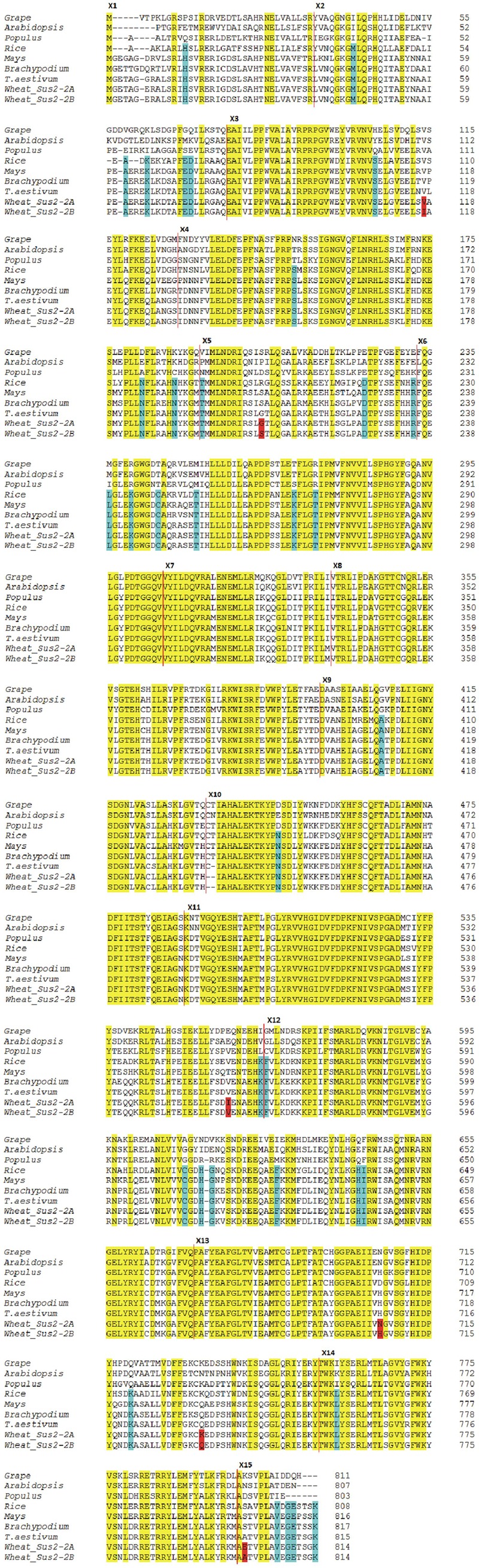
**Dicot vs Monocot SUS2 amino acid sequences.** The derived amino acid sequences of the wheat *Sus2-2A* and *Sus2-2B* genes are compared to amino acid sequences of other plants. The SUS2-2A and SUS2-2B deduced sequences from cv Ciccio are indicated as Wheat SUS2-2A and Wheat SUS2-2B. Amino acid changes between Wheat SUS2-2A and Wheat SUS2-2B are highlighted in red. Amino acid residues conserved in all aligned proteins are shaded in yellow. Amino acids unique to monocots are shaded in blue. Vertical red lines indicate exon boundaries. Exon numbers indicated by Xn are to the left of the exon sequence.

The degree of conservation of amino acid sequences among a selection of plant SUSs is shown in **Figure 4**. The *Sus2-2A* coding sequence from the wheat *cv* Ciccio was used to derive the complete SUS2-2A amino acid sequence. Translation was also carried out for available homologous genes from common wheat, *Brachypodium*, maize, rice, *Populus*, grape, *Arabidopsis*. Positions of exon/intron boundaries are conserved among all plants analyzed. Even if a clear distinctive pattern of differences can be observed between the dicotyledonous and monocotyledonous sequences, all the aligned proteins showed the primary sequence structure of a typical SUS: a cellular target domain (CTD, residues 13–130), an ENOD40 peptide-binding domain (EPBD, residues 160–279), and two domains that comprise the GT-B glycosyltransferase (residues 280–777) (Lairson et al., 2008).

**FIGURE 5:**
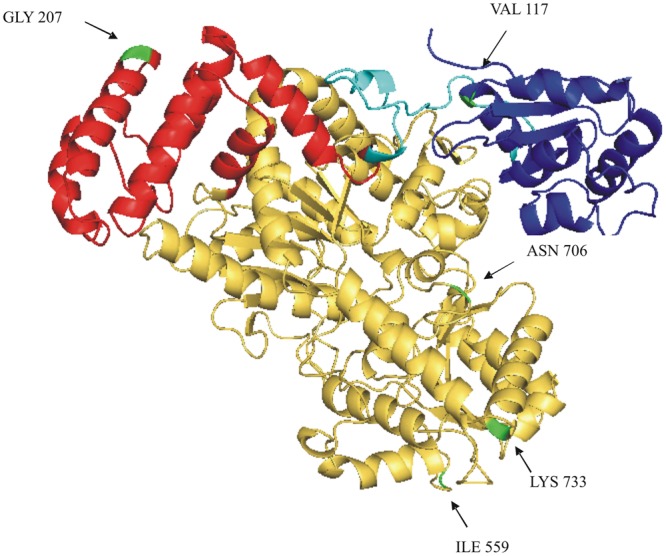
**Ribbon drawing of a SUS2-2A monomer structural model.** A structural model of SUS2-2A, obtained against AtSUS1 protein structure (pdb 3S29C) is shown. The CTD (cellular targeting domain) is colored in blue; the EPBD (ENOD40 peptide-binding domain) is colored in red; the linker region between the CTD and EPDB domains is colored in light blue; the GT-B glycosyltransferase is colored in yellow. The five different amino acids between SUS2-2A and SUS2-2B proteins are colored in green and indicated by arrows.

Six amino acids differ in the comparison of the deduced SUS2-2A and SUS2-2B protein sequences (**Figure 4**). In order to analyze a possible functional implication of the observed differences a three-dimensional model of SUS2 was derived by applying the Phyre 2 One-to-one threading software using the SUS 1 of *A. thaliana* structure (PDB: 3S29C) as reference. Specifically 779 residues (96% of the total SUS2 sequence) were modeled with 100% confidence against the template. The output of the analysis was visualized using the PyMOL software, where the SUS2 protein is represented by a single monomer (**Figure 5**). To maximize the stability of the deduced monomer, the 801 amino acid residue was removed.

## Discussion

The present report describes the identification of the wheat *Sus2* gene sequences in durum wheat cvs Ciccio and Svevo, together with their expression analysis carried out by RT-PCR and activity assays of encoded enzymes, compared to other two different genotypes (Primadur, 5-BIL42), chosen for their differences in important qualitative and quantitative traits, including grain yield components (Blanco et al., 2012; Pasqualone et al., 2015). SUS is a key enzyme in plant sucrose catabolism, uniquely able to mobilize sucrose into different pathways involved in metabolic, structural, and storage functions.

Two different haplotypes have already been identified in common wheat (*TaSus2-2A* and *TaSus2-2B*) that were found significantly associated with one TGW based on field data of the Chinese MCC, Chinese modern cultivars, and NILs evaluated in multiple environments (Jiang et al., 2011; Hou et al., 2014).

In this study, we used the cDNA sequence of *TaSus2* gene (AJ000153.1) to extract from public databases overlapping Roche 454 sequences of wheat (cv Chinese Spring) genome^2^ from public websites. In this way, we were able to assemble a unique genomic sequence, corresponding to a complete *T. aestivum Sus2* gene. Specific PCR primer pairs were then used to identify and characterize the two homoeologous genes in the durum wheat cvs Ciccio and Svevo, two commercial cvs widely grown in Italy and polymorphic for grain yield components and protein content: the first one has a higher grain yield and thousand kernel weight while the second one has a higher grain protein content (GPC) (Blanco et al., 2012). The nucleotide sequences of the two homoeologous genes have the same intron/exon structure with identical SNP differences in both introns and exons, in the genomes of the two cvs, and even in the Chinese Spring genome. On the basis of these observations, it was not possible to genetically map *Sus2* genes in Svevo x Ciccio RIL mapping population. Nevertheless, genetic analyses carried out by Blanco et al. (2012), led to the identification of QTLs for yield components on homoeologous chromosome group 2. In particular, in the centromeric region of chromosome 2B, Jiang et al. (2011) mapped a *TaSus2*-2B gene, close to markers barc18 and wms55, where Blanco et al. (2012) co-localized a grain yield per spike QTL. This result could suggest the involvement of this gene in the phenotypic expression of grain yield.

The amino acid sequence corresponding to the *Sus2* coding sequence from the two durum wheat cvs Ciccio and Svevo (**Figure 4**) was compared to amino acids sequences derived from homologous genes from model and crop species such as common wheat, *Brachypodium*, maize, rice, *Populus*, grape, *Arabidopsis.* Despite the presence of some amino acid differences among sequences, it is clear that some regions are highly conserved in all the examined species, probably corresponding to regions of critical functionality, essential for enzyme activity. Four distinct protein domains, typical of the SUS enzyme, are present: a cellular targeting domain (CTD), an EPBD and a GT-B glycosyltransferase with its Rossmann-fold domain (Lairson et al., 2008). Length and position of these domains are highly conserved in all the species examined, as shown in **Figure 4**. Six amino acids differences were found by comparison of the deduced SUS2-2A and SUS2-2B protein sequences in the two durum wheat cvs Ciccio and Svevo. SUS2-2A protein was modeled against the SUS 1 of *A. thaliana* (AtSus1). The different amino acid residues were analyzed and their possible interactions with residues located at a distance of 8 Å were checked. In SUS2-2B a possible hydrogen bond was found for the Ser residue in position 207 with different amino acids (Arg 204, Ser 205, Thr 208, Gln 210, Arg 214), which is located in the EPBD (residues 160–279), eventually leading to a major rigidity of the domain.

In the present study, the transcriptional levels of the *Sus2-2A* and *Sus2-2B* genes were studied in the wheat genotypes Svevo, Ciccio, Primadur, and 5-BIL42. qRT-PCR revealed a differential pattern for the two genes in the analyzed lines. Significant different expression levels were observed for both *Sus2-2A* and *Sus2-2B* genes in kernels at milk stage with respect to dough stage. Surprisingly, Svevo, Primadur, and 5-BIL42 showed a general higher level of transcription for both genes. Specifically in cv Svevo a significant higher level of *Sus2-2B* transcript compared to *Sus2-2B* of the cv Ciccio was found. These results were apparently in contrast to the enzyme activity and the higher thousand kernel weight previously found. However, a correlation between expressed mRNAs and protein content is not always occurring, as already reported for mammalian cells, yeast and plants (Chen et al., 2002; Liu et al., 2004; Tian et al., 2004; Dembinsky et al., 2007; Foss et al., 2007; Hennig, 2007; Lu et al., 2007; Cho et al., 2008; Rogers et al., 2008). Recent studies have showed that starch biosynthesis is mainly regulated by protein posttranslational modifications, especially by phosphorylation (Tetlow et al., 2008). Moreover, although the roles of a few isoforms have been probed in transgenic and mutants plants, no systematic functional analysis for the gene families has been reported. Mutant plants of *A. thaliana* lacking individual isoforms have shown no obvious growth phenotypes and were not significantly different from wild type plants in starch content under the growth conditions employed.

Obtained data indicated that SUS2 activity, evaluated in the kernel endosperm collected at milk stage, was higher in cv Ciccio than in other cvs analyzed. Specifically, the SUS2 activity in cv Ciccio was 54.0% higher compared to Primadur (**Figure 3**). This might support the view of an involvement of these two enzymes in the metabolic pathway of starch accumulation and then wheat yield.

In order to confirm our hypothesis, future research might involve the production of the recombinant proteins SUS2-2A and SUS2-2B to verify the possible roles of the amino acid variations in protein activity. In this perspective new experiments might address this proposition via specific down or over-expression of the *Sus2* genes by transformation with specific *Sus2* gene constructs. The availability of the *Sus2* gene sequences make this molecular approach possible.

## Author Contributions

MV, IF, AG, AB, LC: conceived and designed the experiments. MV, IF, CL, DN, BG: performed the experiments. MV, AG, AB, GM: contributed reagents/materials/analysis tools. MV, IF, AG, LC: wrote the paper. MV, IF, AG, GM, LC: analyzed the data.

## Conflict of Interest Statement

The authors declare that the research was conducted in the absence of any commercial or financial relationships that could be construed as a potential conflict of interest.

## Footnotes

1 http://www.wheatgenome.org/reference

2 www.cerealsdb.uk.net
