# Supplementary material for: Identification and Characterization of the Sucrose Synthase 2 Gene (Sus2) in Durum Wheat
Source: Front Plant Sci. 2016 Mar 10;7:266. doi: 10.3389/fpls.2016.00266 (PMC4785679; doi:10.3389/fpls.2016.00266)
Supplement: Supplementary file 1 [file Data_Sheet_1.DOCX]

**Supplementary Material**

**Table S1.** **Oligonucleotides used in PCR, RT-PCR, qRT-PCR and 3’-RACE experiments.**

| **PRIMER NAME** | **SEQUENCE (5’-3’)** | **Tm (◦C)** |
| --- | --- | --- |
| Sus2-167for | CGCCCTGAGCCGCATCCACA | 65.5 |
| Sus2-168rev | CGCTCGCCCGCCATTTATTTCTCT | 64.4 |
| Rev1-ciccio | CCTGTGCGCCCCTTAGGAGA | 63.5 |
| FOR-5’prom | CGGAGTTTCTGGAATGCAAAAG | 63 |
| SUS2-REV7 | GATCCAGCTCATACTATAGC | 55.3 |
| SUS2-REV9 | CAAACCGGCAGCCAGTTTAGCCTGC | 67.9 |
| FORA-3RACE | GATCATTGTGAATGGTGTGTC | 55.9 |
| FORB-3RACE | CAGCTGAGATCATTGTGCAC | 57.3 |
| FORA2-3RACE | GTTTCTGGAAGTACGTCTCC | 57.3 |
| FORB2-3RACE | GTCTATGGTTTCTGGAAGTAT | 54.0 |
| FOR-ESII | GAGTACAATGCCGCGATCCC | 61.4 |
| REV-ESVI | GAGCACAGTCGCCCCAACC | 63.1 |
| FOR-ESVI | GCTCAGGCCAATGTCTTGGG | 61.4 |
| REV-ESIX | GCCATCACTGTAGTTTCCAATG | 58.4 |
| FOR-ESX | GCAATGAACCATGCCGACTTC | 59.8 |
| REV-ESXII | CTACCAGGCCAGTCATATTCTTG | 60.6 |
| FOR-ESXII | CCGCAATGGTGAGCTCTACC | 61.4 |
| REV-ESXIII | CTCCCTGCGAGATCTTGTTCC | 61.8 |
| FOR-ESXIII | GGAAGACCCGAGCCACTGG | 63.1 |
| REV-ESXV | CCGAGGTCTCGCCCTCAAC | 63.1 |
| RT-6E-For1 | GGACAATCCCAATGGTGTTC | 68 |
| RT-7E-Rev1 | ATGTCGAGACCTTGCTGCTT | 68 |
| RT-6E-For2 | CTCGGAACAATCCCAATGGT | 68 |
| RT-7E-Rev2 | TGTAATGTCAAGACCTTGCTG | 67 |
| RT-9E-FORA | GCATGTTTGTTAGCTCACAAG | 67 |
| RT-10E-REVA | GGTTCATTGCAATCAGGTCG | 68 |
| RT-9E-FORB | ACCTAGTCGCGTGTTTGTTG | 68 |
| RT-10E-REVB | TTGCAATCAGGTCAGCTGTG | 68 |
| CDC-FOR | CAAATACGCCATCAGGGAGAACATC | 74 |
| CDC-REV | CGCTGCCGAAACCACGAGAC | 74 |
| RLI-FOR | CGATTCAGAGCAGCGTATTGTTG | 72 |
| RLI-REV | AGTTGGTCGGGTCTCTTCTAAATG | 72 |
| ADP-RIF-FOR | GACCACCATCCTCTACAAG | 68 |
| ADP-RIF-REV | AGCAGCACAGCATCAC | 62 |

**Figure S1.** **Genomic sequences of the two *Sus2-2A* and *Sus2-2B* homoeologous genes.** ClustalW alignments of *Sus2-2A* and *Sus2-2B* sequences of the two durum wheat cvs Svevo and Ciccio. Boxed in yellow the initial codon ATG and the stop codon TGA; exons are delimitated by red brackets.

Sus2_2B_Ciccio CGGAGTTTCTGGAATGCAAAAGTAAATCTAGCAGGTTTTTTT--CTTCCTATTTTATGCA 58

Sus2_2B_Svevo CGGAGTTTCTGGAATGCAAAAGTAAATCTAGCAGGTTTTTTT--CTTCCTATTTTATGCA 58

Sus2_2A_Ciccio CGGAGTTTCTGGAATGCAAAAGTAAATCTAGCAGTTTTTTTTTGCTTCCTATTTTACGCA 60

Sus2_2A_Svevo CGGAGTTTCTGGAATGCAAAAGTAAATCTAGCAGTTTTTTTTTGCTTCCTATTTTACGCA 60

********************************** ******* ************ ***

Sus2_2B_Ciccio ACTTGTTAAAGGGGCTATTAAAGTTTT------ATCAAGGCCAGTAATTAAGAGTTACAG 112

Sus2_2B_Svevo ACTTGTTAAAGGGGCTATTAAAGTTTT------ATCAAGGCCAGTAATTAAGAGTTACAG 112

Sus2_2A_Ciccio ACTTGTTAAA---GCTATTAACTTTTCCTGCAGAGCAAAGCTATTAACTCTGTACTCCAA 117

Sus2_2A_Svevo ACTTGTTAAA---GCTATTAACTTTTCCTGCAGAGCAAAGCTATTAACTCTGTACTCCAA 117

********** ******** *** * *** ** * *** * * * **

Sus2_2B_Ciccio TGGTTCTC----ACTAATC-CTACATAAGCTTCTTGCCTTGCGGATAATGTGCATGTTTT 167

Sus2_2B_Svevo TGGTTCTC----ACTAATC-CTACATAAGCTTCTTGCCTTGCGGATAATGTGCATGTTTT 167

Sus2_2A_Ciccio AGTTTTTCCAAGGCCAGTAATTAATTAAGCTTCTTGCCTTGTGGATCATGTGCATGTCAT 177

Sus2_2A_Svevo AGTTTTTCCAAGGCCAGTAATTAATTAAGCTTCTTGCCTTGTGGATCATGTGCATGTCAT 177

* ** ** * * * ** **************** **** ********** *

Sus2_2B_Ciccio TGTATTACTACTGTACTAAATTAGCTGATCCGTTTCTATTTATTTGCATGCTCTCTACTG 227

Sus2_2B_Svevo TGTATTACTACTGTACTAAATTAGCTGATCCGTTTCTATTTATTTGCATGCTCTCTACTG 227

Sus2_2A_Ciccio TGTATAA------TACTA------------------------------------------ 189

Sus2_2A_Svevo TGTATAA------TACTA------------------------------------------ 189

***** * *****

Sus2_2B_Ciccio GAAAAGTGGAGAGCCTGCCCGGCAATAGTCCCGTGCTCCTGTGCTCACTCATATTTTGGT 287

Sus2_2B_Svevo GAAAAGTGGAGAGCCTGCCCGGCAATAGTCCCGTGCTCCTGTGCTCACTCATATTTTGGT 287

Sus2_2A_Ciccio -------GGAGTAC---------------------------------------------- 196

Sus2_2A_Svevo -------GGAGTAC---------------------------------------------- 196

**** *

Sus2_2B_Ciccio CAGTGTTAATTACTTGATCTGTTTCTAGTATCTGCATGCCCTTTACTGGAAAACTGGAGA 347

Sus2_2B_Svevo CAGTGTTAATTACTTGATCTGTTTCTAGTATCTGCATGCCCTTTACTGGAAAACTGGAGA 347

Sus2_2A_Ciccio -----TAAATTACTTGATCCGTTTCTATTATCTGCATGTCCTCCATTGGAAAACTGGAGA 251

Sus2_2A_Svevo -----TAAATTACTTGATCCGTTTCTATTATCTGCATGTCCTCCATTGGAAAACTGGAGA 251

* ************ ******* ********** *** * **************

Sus2_2B_Ciccio GCCTGCCCGGCAATAGTTCTGTCTTCTGTGCTCCTGCGCTCACTCATATACTTTGGTCAG 407

Sus2_2B_Svevo GCCTGCCCGGCAATAGTTCTGTCTTCTGTGCTCCTGCGCTCACTCATATACTTTGGTCAG 407

Sus2_2A_Ciccio GCCTGCCCGGCAATAGTTCCGT-------GCTCCTGTGCTCACTCATATACTTTGGTCAG 304

Sus2_2A_Svevo GCCTGCCCGGCAATAGTTCCGT-------GCTCCTGTGCTCACTCATATACTTTGGTCAG 304

******************* ** ******* ***********************

Sus2_2B_Ciccio TTTTAATTAGCTTGCCAAGCCACGCAAGGAACGACTAGGATACATCTCACGCTAATCCCA 467

Sus2_2B_Svevo TTTTAATTAGCTTGCCAAGCCACGCAAGGAACGACTAGGATACATCTCACGCTAATCCCA 467

Sus2_2A_Ciccio TTTTAATTAGCTTGCCAAGCCACGCAAGAAACGGCTACAATACATCTCACGCTAATCCCA 364

Sus2_2A_Svevo TTTTAATTAGCTTGCCAAGCCACGCAAGAAACGGCTACAATACATCTCACGCTAATCCCA 364

**************************** **** *** *********************

Sus2_2B_Ciccio TGATCTGCTTCCCAAAGCACAACCTTCTAGGTACTCTTGCACGTCACGCCAACCTTTCTT 527

Sus2_2B_Svevo TGATCTGCTTCCCAAAGCACAACCTTCTAGGTACTCTTGCACGTCACGCCAACCTTTCTT 527

Sus2_2A_Ciccio TGATCTGGTTCCCAAAGCACAGGCTTCTAGGCACTCTTGCACGCCACGCCAACCTTTCTT 424

Sus2_2A_Svevo TGATCTGGTTCCCAAAGCACAGGCTTCTAGGCACTCTTGCACGCCACGCCAACCTTTCTT 424

******* ************* ******** *********** ****************

Sus2_2B_Ciccio CTTCCTTTGGCTCTATTTATCAGTAGAGCTCATGCATCGTTGCTACTAGGACAGAGCGCC 587

Sus2_2B_Svevo CTTCCTTTGGCTCTATTTATCAGTAGAGCTCATGCATCGTTGCTACTAGGACAGAGCGCC 587

Sus2_2A_Ciccio CTTCCTCCGGCTCTATATATCAGTAGAGCTCATGCATCGTTGCC---AGGACAGAGCGCC 481

Sus2_2A_Svevo CTTCCTCCGGCTCTATATATCAGTAGAGCTCATGCATCGTTGCC---AGGACAGAGCGCC 481

****** ******** ************************** *************

Sus2_2B_Ciccio CTGTTGTCTAGGAGGAGGCTTCACTGTTTCTGCTGTTCAGGTTTACATGTCTGTTTCCGC 647

Sus2_2B_Svevo CTGTTGTCTAGGAGGAGGCTTCACTGTTTCTGCTGTTCAGGTTTACATGTCTGTTTCCGC 647

Sus2_2A_Ciccio CTGTTGTGTGGGAGGAGGCTCCACTGTTTCTGCTGTTCAGATTTACATGACTGTTTCCGC 541

Sus2_2A_Svevo CTGTTGTGTGGGAGGAGGCTCCACTGTTTCTGCTGTTCAGATTTACATGACTGTTTCCGC 541

******* * ********** ******************* ******** **********

Sus2_2B_Ciccio TGTTCAGGTTTGAGGACAGCAATGGGGGAGACTGCCGGAGAGCGCGCCCTGAGCCGCATC 707

Sus2_2B_Svevo TGTTCAGGTTTGAGGACAGCAATGGGGGAGACTGCCGGAGAGCGCGCCCTGAGCCGCATC 707

Sus2_2A_Ciccio TGTTCAGGTTTGAGGACAGCAATGGGGGAGACTGCCGGAGAGCGCGCCCTGAGCCGCATC 601

Sus2_2A_Svevo TGTTCAGGTTTGAGGACAGCAATGGGGGAGACTGCCGGAGAGCGCGCCCTGAGCCGCATC 601

************************************************************

Sus2_2B_Ciccio CACAGCGTGAGGGAGCGCATCGGCGATTCCCTCTCCGCGCACACCAATGAGCTTGTCGCC 767

Sus2_2B_Svevo CACAGCGTGAGGGAGCGCATCGGCGATTCCCTCTCCGCGCACACCAATGAGCTTGTCGCC 767

Sus2_2A_Ciccio CACAGCGTGAGGGAGCGCATCGGTGATTCCCTCTCTGCGCACACCAATGAGCTCGTCGCC 661

Sus2_2A_Svevo CACAGCGTGAGGGAGCGCATCGGTGATTCCCTCTCTGCGCACACCAATGAGCTCGTCGCC 661

*********************** *********** ***************** ******

Sus2_2B_Ciccio GTCTTCTCAAGGTCTGTCCTTGATCTTTTGCAGGCTAAACTGGCTGCCGGTTTGTAGAAA 827

Sus2_2B_Svevo GTCTTCTCAAGGTCTGTCCTTGATCTTTTGCAGGCTAAACTGGCTGCCGGTTTGTAGAAA 827

Sus2_2A_Ciccio GTCTTCTCAAGGTCTGTCCCTGATCTTTTGCAGGCTAGACTGGCTGCCAATTTGTACAAA 721

Sus2_2A_Svevo GTCTTCTCAAGGTCTGTCCCTGATCTTTTGCAGGCTAGACTGGCTGCCAATTTGTACAAA 721

******************* ***************** ********** ****** ***

Sus2_2B_Ciccio TCTCTGCATATAAGAATGATATGGTTAAGCGATGAATTATGGCTGCCTTGTGTATTTCTA 887

Sus2_2B_Svevo TCTCTGCATATAAGAATGATATGGTTAAGCGATGAATTATGGCTGCCTTGTGTATTTCTA 887

Sus2_2A_Ciccio TCTCTGCATGTGAG----ACACGGTTAAGCGATGAATTATGGTTGCCTTGCGTATTTCTA 777

Sus2_2A_Svevo TCTCTGCATGTGAG----ACACGGTTAAGCGATGAATTATGGTTGCCTTGCGTATTTCTA 777

********* * ** * * ******************** ******* *********

Sus2_2B_Ciccio TGTTATGCTATAGTATGAGCTGGATCAATGGCTTTTTTCTTTAAAAAGTCTTGTTACTAA 947

Sus2_2B_Svevo TGTTATGCTATAGTATGAGCTGGATCAATGGCTTTTTTCTTTAAAAAGTCTTGTTACTAA 947

Sus2_2A_Ciccio TGTCATGCTATAGTATGAGCTGGATCAATGATTTTTTTCTTTACAAAAT-TTGT-ACCAA 835

Sus2_2A_Svevo TGTCATGCTATAGTATGAGCTGGATCAATGATTTTTTTCTTTACAAAAT-TTGT-ACCAA 835

*** ************************** *********** *** * **** ** **

Sus2_2B_Ciccio GGACCATTAAAAGAACTGATGATGAAGTTTCAACAATTGTTCCTGCATTTCTATATAGTA 1007

Sus2_2B_Svevo GGACCATTAAAAGAACTGATGATGAAGTTTCAACAATTGTTCCTGCATTTCTATATAGTA 1007

Sus2_2A_Ciccio GGGCCATTAAAAGAACTGATGATGAAGTTTCAACAATTGTTCCTGCATTTCTATATAATA 895

Sus2_2A_Svevo GGGCCATTAAAAGAACTGATGATGAAGTTTCAACAATTGTTCCTGCATTTCTATATAATA 895

** ****************************************************** **

Sus2_2B_Ciccio CTGCTCCTTCTATTTGTCTTTTAACTTCTTTCTTAAT-CTAGTTGTATTTATACTATAAC 1066

Sus2_2B_Svevo CTGCTCCTTCTATTTGTCTTTTAACTTCTTTCTTAAT-CTAGTTGTATTTATACTATAAC 1066

Sus2_2A_Ciccio CCGCTCCTTCCATTTGTCTTTTAACTTCTTTCTTAATACTAGTTGTATTCATACTATAAC 955

Sus2_2A_Svevo CCGCTCCTTCCATTTGTCTTTTAACTTCTTTCTTAATACTAGTTGTATTCATACTATAAC 955

* ******** ************************** *********** **********

Sus2_2B_Ciccio AAAGTACCTGAACATTTTCTTACTGCTACAATTGTTACCATACTTTGTTCTTCAAGTAAC 1126

Sus2_2B_Svevo AAAGTACCTGAACATTTTCTTACTGCTACAATTGTTACCATACTTTGTTCTTCAAGTAAC 1126

Sus2_2A_Ciccio AAAGTACCTGAACATTTTCTTACTGCTACAATTGTTACCACACTTTGT---TCAAGTAAC 1012

Sus2_2A_Svevo AAAGTACCTGAACATTTTCTTACTGCTACAATTGTTACCACACTTTGT---TCAAGTAAC 1012

**************************************** ******* *********

Sus2_2B_Ciccio GTTTGTTGATAGATCTGACCCACTGGCTGTTACATGATAACTTGTTCAGGCTTGTTAACC 1186

Sus2_2B_Svevo GTTTGTTGATAGATCTGACCCACTGGCTGTTACATGATAACTTGTTCAGGCTTGTTAACC 1186

Sus2_2A_Ciccio GTTTGTTGATAGATCTGACCCACCTGCTGTTACACGATAACTTGTTCAGGCTTGTTAACC 1072

Sus2_2A_Svevo GTTTGTTGATAGATCTGACCCACCTGCTGTTACACGATAACTTGTTCAGGCTTGTTAACC 1072

*********************** ********* *************************

Sus2_2B_Ciccio AAGGAAAGGGGATGCTGCAGCCCCATCAGATAACTGCTGAGTACAATGCCGCGATCCCAG 1246

Sus2_2B_Svevo AAGGAAAGGGGATGCTGCAGCCCCATCAGATAACTGCTGAGTACAATGCCGCGATCCCAG 1246

Sus2_2A_Ciccio AAGGAAAGGGGATGCTGCAGCCCCATCAGATCACTGCTGAGTACAATGCCGCGATCCCTG 1132

Sus2_2A_Svevo AAGGAAAGGGGATGCTGCAGCCCCATCAGATCACTGCTGAGTACAATGCCGCGATCCCTG 1132

******************************* ************************** *

Sus2_2B_Ciccio AGGCCGAGCGTGAGAAGCTCAAGGACACCGCCTTTGAGGATCTCCTAAGGGGCGCACAGG 1306

Sus2_2B_Svevo AGGCCGAGCGTGAGAAGCTCAAGGACACCGCCTTTGAGGATCTCCTAAGGGGCGCACAGG 1306

Sus2_2A_Ciccio AGGCCGAGCGCGAGAAGCTCAAGGACACCGCCTTTGAGGATCTCCTAAGGGGCGCACAGG 1192

Sus2_2A_Svevo AGGCCGAGCGCGAGAAGCTCAAGGACACCGCCTTTGAGGATCTCCTAAGGGGCGCACAGG 1192

********** *************************************************

Sus2_2B_Ciccio TTTGCACCACCAAAACTCACTGCAGTGTCATTCAAGTGATGTTCAGCTCTTGATCTGTTG 1366

Sus2_2B_Svevo TTTGCACCACCAAAACTCACTGCAGTGTCATTCAAGTGATGTTCAGCTCTTGATCTGTTG 1366

Sus2_2A_Ciccio TTTGCACCACCAAAACGCACTGCACTGTCATTCCAGTGATGTTCAGCTCTTGATCTGTTG 1252

Sus2_2A_Svevo TTTGCACCACCAAAACGCACTGCACTGTCATTCCAGTGATGTTCAGCTCTTGATCTGTTG 1252

**************** ******* ******** **************************

Sus2_2B_Ciccio GTTTCTCATACAGGAGGCAATTGTCATCCCTCCATGGGTTGCTCTCGCCATCCGGCCAAG 1426

Sus2_2B_Svevo GTTTCTCATACAGGAGGCAATTGTCATCCCTCCATGGGTTGCTCTCGCCATCCGGCCAAG 1426

Sus2_2A_Ciccio GTTTCTCATATAGGAGGCAATTGTCATCCCTCCATGGGTTGCTCTCGCCATCCGGCCAAG 1312

Sus2_2A_Svevo GTTTCTCATATAGGAGGCAATTGTCATCCCTCCATGGGTTGCTCTCGCCATCCGGCCAAG 1312

********** *************************************************

Sus2_2B_Ciccio GCCCGGCGTCTGGGAGTATGTGAGGGTCAATGTGAGCGAGCTTGGTGTTGAGGAGTTAAG 1486

Sus2_2B_Svevo GCCCGGCGTCTGGGAGTATGTGAGGGTCAATGTGAGCGAGCTTGGTGTTGAGGAGTTAAG 1486

Sus2_2A_Ciccio GCCCGGCGTCTGGGAGTATGTGAGGGTCAATGTGAGCGAGCTTGGTGTTGAGGAGTTGAG 1372

Sus2_2A_Svevo GCCCGGCGTCTGGGAGTATGTGAGGGTCAATGTGAGCGAGCTTGGTGTTGAGGAGTTGAG 1372

********************************************************* **

Sus2_2B_Ciccio CATCGCTGAGTATTTGCAGTTCAAGGAACAACTGGCGAATGGAAGGTATCTGTGATTTTG 1546

Sus2_2B_Svevo CATCGCTGAGTATTTGCAGTTCAAGGAACAACTGGCGAATGGAAGGTATCTGTGATTTTG 1546

Sus2_2A_Ciccio CGTCGCTGAGTATTTGCAGTTCAAGGAACAACTGGCGAATGGAAGGTATCTGTGGTTTTG 1432

Sus2_2A_Svevo CGTCGCTGAGTATTTGCAGTTCAAGGAACAACTGGCGAATGGAAGGTATCTGTGGTTTTG 1432

* **************************************************** *****

Sus2_2B_Ciccio TGAAATCATTAAGATTCAAGAGTCCACCTTATACCTTAGTTTT-ATCATACAACGCTTCT 1605

Sus2_2B_Svevo TGAAATCATTAAGATTCAAGAGTCCACCTTATACCTTAGTTTT-ATCATACAACGCTTCT 1605

Sus2_2A_Ciccio TGAAATTATTAAGATTCAAGAAGTCAGATTATACCTTAGTTTTTATTATATAACACTTCT 1492

Sus2_2A_Svevo TGAAATTATTAAGATTCAAGAAGTCAGATTATACCTTAGTTTTTATTATATAACACTTCT 1492

****** ************** ** *************** ** *** *** *****

Sus2_2B_Ciccio TCTGTTCAAATTGCAGCATCGATAACAACTTTGTGCTTGAGCTGGACTTTGAGCCATTCA 1665

Sus2_2B_Svevo TCTGTTCAAATTGCAGCATCGATAACAACTTTGTGCTTGAGCTGGACTTTGAGCCATTCA 1665

Sus2_2A_Ciccio TCTGTTCAAATTGCAGCATCGATAACAACTTTGTGCTTGAGCTGGACTTTGAGCCATTCA 1552

Sus2_2A_Svevo TCTGTTCAAATTGCAGCATCGATAACAACTTTGTGCTTGAGCTGGACTTTGAGCCATTCA 1552

************************************************************

Sus2_2B_Ciccio ACGCCTCCTTCCCTCGCCCATCGCTGTCGAAGTCCATTGGCAACGGTGTGCAGTTTCTGA 1725

Sus2_2B_Svevo ACGCCTCCTTCCCTCGCCCATCGCTGTCGAAGTCCATTGGCAACGGTGTGCAGTTTCTGA 1725

Sus2_2A_Ciccio ACGCCTCCTTCCCGCGCCCATCGCTGTCGAAGTCCATTGGCAACGGTGTGCAGTTCCTGA 1612

Sus2_2A_Svevo ACGCCTCCTTCCCGCGCCCATCGCTGTCGAAGTCCATTGGCAACGGTGTGCAGTTCCTGA 1612

************* ***************************************** ****

Sus2_2B_Ciccio ACAGGCACTTGTCATCGAAGCTGTTCCATGACAAGGAGAGCATGTACCCATTGCTCAACT 1785

Sus2_2B_Svevo ACAGGCACTTGTCATCGAAGCTGTTCCATGACAAGGAGAGCATGTACCCATTGCTCAACT 1785

Sus2_2A_Ciccio ACAGGCACTTGTCATCGAAGCTGTTCCATGACAAGGAGAGCATGTACCCATTGCTCAACT 1672

Sus2_2A_Svevo ACAGGCACTTGTCATCGAAGCTGTTCCATGACAAGGAGAGCATGTACCCATTGCTCAACT 1672

************************************************************

Sus2_2B_Ciccio TCCTTCGCGCGCACAACTACAAGGGGATGGTAGGTTACACTCTCCAGTGTCTGGCTCTGT 1845

Sus2_2B_Svevo TCCTTCGCGCGCACAACTACAAGGGGATGGTAGGTTACACTCTCCAGTGTCTGGCTCTGT 1845

Sus2_2A_Ciccio TCCTTCGCGCGCACAACTACAAGGGGATGGTAGGTTACACTCTCCAGTGTCTTGCTTTGT 1732

Sus2_2A_Svevo TCCTTCGCGCGCACAACTACAAGGGGATGGTAGGTTACACTCTCCAGTGTCTTGCTTTGT 1732

**************************************************** *** ***

Sus2_2B_Ciccio AGATTGGATCATTTGGCTTTGTAGATTGGATCATTCAACTATATCTTTGCTGAAGGTTCT 1905

Sus2_2B_Svevo AGATTGGATCATTTGGCTTTGTAGATTGGATCATTCAACTATATCTTTGCTGAAGGTTCT 1905

Sus2_2A_Ciccio AGATTGGATCGTTTGGCTTTGTAGATTGGATGATTCGACTATATCTTTGCTGAAGGTTCT 1792

Sus2_2A_Svevo AGATTGGATCGTTTGGCTTTGTAGATTGGATGATTCGACTATATCTTTGCTGAAGGTTCT 1792

********** ******************** **** ***********************

Sus2_2B_Ciccio CTAGAAAAACTATATCTTTGTTGTTGAA--------GGCTTACATCATTTGTTTATAGCA 1957

Sus2_2B_Svevo CTAGAAAAACTATATCTTTGTTGTTGAA--------GGCTTACATCATTTGTTTATAGCA 1957

Sus2_2A_Ciccio ATAGAAA--CTACATCTTTGTTGTTGAAAACTGACAGGCTTACATCATTTGTTAATGGCA 1850

Sus2_2A_Svevo ATAGAAA--CTACATCTTTGTTGTTGAAAACTGACAGGCTTACATCATTTGTTAATGGCA 1850

****** *** *************** ***************** ** ***

Sus2_2B_Ciccio TGTCAATCTCTTAGATATATCTAAAAGCAGCATATGAGCATACCCAGCCTGTAAGAAAAT 2017

Sus2_2B_Svevo TGTCAATCTCTTAGATATATCTAAAAGCAGCATATGAGCATACCCAGCCTGTAAGAAAAT 2017

Sus2_2A_Ciccio TGGCGATCTCTTAGATATATCTAAAGGCAGCATATGAGCATAGTCAGCCTGCAAGAAAAT 1910

Sus2_2A_Svevo TGGCGATCTCTTAGATATATCTAAAGGCAGCATATGAGCATAGTCAGCCTGCAAGAAAAT 1910

** * ******************** **************** ******* ********

Sus2_2B_Ciccio ATAGATACAATCT-ACTTAATGTGTTGGGCATTTGCCCTGATGATATGTGCATTT-GTAA 2075

Sus2_2B_Svevo ATAGATACAATCT-ACTTAATGTGTTGGGCATTTGCCCTGATGATATGTGCATTT-GTAA 2075

Sus2_2A_Ciccio ATAGACACGATCTTACTTAATGTGTCAGGCATTTGCCCTGATGATATGTGCATTTTGTAT 1970

Sus2_2A_Svevo ATAGACACGATCTTACTTAATGTGTCAGGCATTTGCCCTGATGATATGTGCATTTTGTAT 1970

***** ** **** *********** **************************** ***

Sus2_2B_Ciccio GTAAAAAAAAAGTTTTAGAACAAATTCTTTAAG-AAGTGCAAAAGCCTGATTGCTATCTA 2134

Sus2_2B_Svevo GTAAAAAAAAAGTTTTAGAACAAATTCTTTAAG-AAGTGCAAAAGCCTGATTGCTATCTA 2134

Sus2_2A_Ciccio GTAAAAAAA--GTTTTAGAACAAATTCTTTAAGTAAGTGCAAAAGCCTGATTGCTATCTA 2028

Sus2_2A_Svevo GTAAAAAAA--GTTTTAGAACAAATTCTTTAAGTAAGTGCAAAAGCCTGATTGCTATCTA 2028

********* ********************** **************************

Sus2_2B_Ciccio TGAGCCAACTGAAAGTTTAAACTGGAACTATTAGACCTGTTGTTTAATCACAATGAGCAT 2194

Sus2_2B_Svevo TGAGCCAACTGAAAGTTTAAACTGGAACTATTAGACCTGTTGTTTAATCACAATGAGCAT 2194

Sus2_2A_Ciccio TGAGCCAACTGAAAGTTTAAACTGGAACTACCATACCTGCTGCTTAATCATGACCAGCAT 2088

Sus2_2A_Svevo TGAGCCAACTGAAAGTTTAAACTGGAACTACCATACCTGCTGCTTAATCATGACCAGCAT 2088

****************************** * ***** ** ******* * *****

Sus2_2B_Ciccio GTAATATTTTCTTTCTTTCTTCAGACCATGATGTTGAACGACAGAATTCGGAGTCTCAGT 2254

Sus2_2B_Svevo GTAATATTTTCTTTCTTTCTTCAGACCATGATGTTGAACGACAGAATTCGGAGTCTCAGT 2254

Sus2_2A_Ciccio GTAATATTTTCTTTCTTTCTTCAGACCATGATGTTGAACGACAGGATTCGCAGTCTCGGT 2148

Sus2_2A_Svevo GTAATATTTTCTTTCTTTCTTCAGACCATGATGTTGAACGACAGGATTCGCAGTCTCGGT 2148

******************************************** ***** ****** **

Sus2_2B_Ciccio ACCCTCCAAGGTGCACTCAGGAAGGCAGAGACACATCTGTCAGGCCTTCCAGCTGACACC 2314

Sus2_2B_Svevo ACCCTCCAAGGTGCACTCAGGAAGGCAGAGACACATCTGTCAGGCCTTCCAGCTGACACC 2314

Sus2_2A_Ciccio ACCCTCCAAGGTGCACTCAGGAAGGCAGAGACACATCTGTCAGGCCTTCCAGCTGACACC 2208

Sus2_2A_Svevo ACCCTCCAAGGTGCACTCAGGAAGGCAGAGACACATCTGTCAGGCCTTCCAGCTGACACC 2208

************************************************************

Sus2_2B_Ciccio CCTTACTCGGAGTTCCACCACCGGTACTGTATATAATCATCATACACATGATCATTGAAA 2374

Sus2_2B_Svevo CCTTACTCGGAGTTCCACCACCGGTACTGTATATAATCATCATACACATGATCATTGAAA 2374

Sus2_2A_Ciccio CCTTACTCAGAGTTCCACCACCGGTACTGTATATAATCATCATACACATGATCACTGAAA 2268

Sus2_2A_Svevo CCTTACTCAGAGTTCCACCACCGGTACTGTATATAATCATCATACACATGATCACTGAAA 2268

******** ********************************************* *****

Sus2_2B_Ciccio CCCTTATTGCTCTCAACAAAGAACTAAAATCGTTGCGTATCAATTTTTTCGTTGCATAAT 2434

Sus2_2B_Svevo CCCTTATTGCTCTCAACAAAGAACTAAAATCGTTGCGTATCAATTTTTTCGTTGCATAAT 2434

Sus2_2A_Ciccio TCCTTATTGTTCTCAACAAAGAACTAAAATCGTTGC-------------------ATAAT 2309

Sus2_2A_Svevo TCCTTATTGTTCTCAACAAAGAACTAAAATCGTTGC-------------------ATAAT 2309

******** ************************** *****

Sus2_2B_Ciccio TCAGGTTCCAGGAACTTGGTCTGGAGAAAGGTTGGGGCGACTGTGCTCAGCGTGCGAGTG 2494

Sus2_2B_Svevo TCAGGTTCCAGGAACTTGGTCTGGAGAAAGGTTGGGGCGACTGTGCTCAGCGTGCGAGTG 2494

Sus2_2A_Ciccio TCAGGTTCCAGGAACTTGGTTTGGAGAAAGGTTGGGGCGACTGTGCTCAGCGTGCGAGCG 2369

Sus2_2A_Svevo TCAGGTTCCAGGAACTTGGTTTGGAGAAAGGTTGGGGCGACTGTGCTCAGCGTGCGAGCG 2369

******************** ************************************* *

Sus2_2B_Ciccio AGACTATCCACCTTCTCTTGGACCTTCTCGAGGCCCCTGATCCATCCTCCTTGGAGAAGT 2554

Sus2_2B_Svevo AGACTATCCACCTTCTCTTGGACCTTCTCGAGGCCCCTGATCCATCCTCCTTGGAGAAGT 2554

Sus2_2A_Ciccio AGACTATCCACCTTCTCTTGGACCTTCTCGAGGCCCCTGATCCATCCTCCTTGGAGAAGT 2429

Sus2_2A_Svevo AGACTATCCACCTTCTCTTGGACCTTCTCGAGGCCCCTGATCCATCCTCCTTGGAGAAGT 2429

************************************************************

Sus2_2B_Ciccio TCCTCGGAACAATCCCAATGGTGTTCAATGTTGTTATCCTCTCTCCTCATGGTTACTTTG 2614

Sus2_2B_Svevo TCCTCGGAACAATCCCAATGGTGTTCAATGTTGTTATCCTCTCTCCTCATGGTTACTTTG 2614

Sus2_2A_Ciccio TCCTCGGGACAATCCCAATGGTGTTCAATGTCGTTATCCTCTCTCCTCATGGTTACTTTG 2489

Sus2_2A_Svevo TCCTCGGGACAATCCCAATGGTGTTCAATGTCGTTATCCTCTCTCCTCATGGTTACTTTG 2489

******* *********************** ****************************

Sus2_2B_Ciccio CTCAGGCCAATGTCTTGGGGTACCCTGATACTGGTGGACAGGTAGAATCCTCTACCCCAC 2674

Sus2_2B_Svevo CTCAGGCCAATGTCTTGGGGTACCCTGATACTGGTGGACAGGTAGAATCCTCTACCCCAC 2674

Sus2_2A_Ciccio CTCAGGCCAATGTCTTGGGGTACCCTGATACTGGTGGACAGGTAGAATCCCCTACATCAT 2549

Sus2_2A_Svevo CTCAGGCCAATGTCTTGGGGTACCCTGATACTGGTGGACAGGTAGAATCCCCTACATCAT 2549

************************************************** **** **

Sus2_2B_Ciccio TTTTGACAGCTTGACATATTTCCTCTTGATAAACTGAACACCAGGAAATATTTATTATCA 2734

Sus2_2B_Svevo TTTTGACAGCTTGACATATTTCCTCTTGATAAACTGAACACCAGGAAATATTTATTATCA 2734

Sus2_2A_Ciccio TTTTGACAGTTTGACATATTTCCTCTTGATAAAATGAACACTATGAAATCTTTGTTATCA 2609

Sus2_2A_Svevo TTTTGACAGTTTGACATATTTCCTCTTGATAAAATGAACACTATGAAATCTTTGTTATCA 2609

********* *********************** ******* * ***** *** ******

Sus2_2B_Ciccio CCAATTTTACTATGTCATGGCA---CACAGATTGTCTACATTTTGGACCAAGTCCGTGCT 2791

Sus2_2B_Svevo CCAATTTTACTATGTCATGGCA---CACAGATTGTCTACATTTTGGACCAAGTCCGTGCT 2791

Sus2_2A_Ciccio CCAATTATCTTATGTCATGGCAACCCATAGGTTGTTTACATTTTGGACCAAGTCCGTGCT 2669

Sus2_2A_Svevo CCAATTATCTTATGTCATGGCAACCCATAGGTTGTTTACATTTTGGACCAAGTCCGTGCT 2669

****** * ************ ** ** **** ************************

Sus2_2B_Ciccio ATGGAGAATGAGATGCTGTTGAGAATCAAGCAGCAAGGTCTTGACATTACACCAAAGATT 2851

Sus2_2B_Svevo ATGGAGAATGAGATGCTGTTGAGAATCAAGCAGCAAGGTCTTGACATTACACCAAAGATT 2851

Sus2_2A_Ciccio ATGGAGAATGAGATGCTGTTGAGAATCAAGCAGCAAGGTCTCGACATTACACCAAAGATT 2729

Sus2_2A_Svevo ATGGAGAATGAGATGCTGTTGAGAATCAAGCAGCAAGGTCTCGACATTACACCAAAGATT 2729

***************************************** ******************

Sus2_2B_Ciccio CTAATAGTAAGTTTAGTACCCCCAATATGATCGAATATGAACTCTCTATTTTAA------ 2905

Sus2_2B_Svevo CTAATAGTAAGTTTAGTACCCCCAATATGATCGAATATGAACTCTCTATTTTAA------ 2905

Sus2_2A_Ciccio CTAATTGTAAGTTTAGTACC--CAACATGATCGAATATGAGCTCTCTGTTATAAACTTAT 2787

Sus2_2A_Svevo CTAATTGTAAGTTTAGTACC--CAACATGATCGAATATGAGCTCTCTGTTATAAACTTAT 2787

***** ************** *** ************** ****** ** ***

Sus2_2B_Ciccio --TTGATCCCTAATGAGGTATCTTTGTCCTGACATAGGTCACCAGGTTGCTCCCTGATGC 2963

Sus2_2B_Svevo --TTGATCCCTAATGAGGTATCTTTGTCCTGACATAGGTCACCAGGTTGCTCCCTGATGC 2963

Sus2_2A_Ciccio AATTGATTCCTAATGAGGTCTCTTTGGCCTTACATAGGTCACCAGGTTGCTCCCTGATGC 2847

Sus2_2A_Svevo AATTGATTCCTAATGAGGTCTCTTTGGCCTTACATAGGTCACCAGGTTGCTCCCTGATGC 2847

***** *********** ****** *** *****************************

Sus2_2B_Ciccio ACATGGCACCACCTGTGGCCAGCGCCTTGAGAAGGTCCTTGGCACCGAGCACACCCACAT 3023

Sus2_2B_Svevo ACATGGCACCACCTGTGGCCAGCGCCTTGAGAAGGTCCTTGGCACCGAGCACACCCACAT 3023

Sus2_2A_Ciccio ACATGGCACCACCTGTGGCCAGCGCCTCGAGAAGGTCCTTGGCACCGAGCACACCCACAT 2907

Sus2_2A_Svevo ACATGGCACCACCTGTGGCCAGCGCCTCGAGAAGGTCCTTGGCACCGAGCACACCCACAT 2907

*************************** ********************************

Sus2_2B_Ciccio CCTGCGTGTGCCATTCAAAACAGAAGATGGTATTGTTCGCAAATGGATCTCCCGCTTTGA 3083

Sus2_2B_Svevo CCTGCGTGTGCCATTCAAAACAGAAGATGGTATTGTTCGCAAATGGATCTCCCGCTTTGA 3083

Sus2_2A_Ciccio CCTGCGTGTGCCATTCAAAACAGAAGATGGTATTGTTCGCAAATGGATCTCGCGTTTTGA 2967

Sus2_2A_Svevo CCTGCGTGTGCCATTCAAAACAGAAGATGGTATTGTTCGCAAATGGATCTCGCGTTTTGA 2967

*************************************************** ** *****

Sus2_2B_Ciccio AGTCTGGCCTTACCTGGAAGCTTACACTGATGTATGCATTTCACTTTCCAGCAGGTTGTT 3143

Sus2_2B_Svevo AGTCTGGCCTTACCTGGAAGCTTACACTGATGTATGCATTTCACTTTCCAGCAGGTTGTT 3143

Sus2_2A_Ciccio AGTCTGGCCTTACCTGGAAGCTTACACTGATGTATGCATTTCACTTTCCAGCAGGTTGTT 3027

Sus2_2A_Svevo AGTCTGGCCTTACCTGGAAGCTTACACTGATGTATGCATTTCACTTTCCAGCAGGTTGTT 3027

************************************************************

Sus2_2B_Ciccio TGTAGTGTTTCATTAGAATAGCAATCCATCATTTTTTTCATGTTGCTTTCAGGATGTGGC 3203

Sus2_2B_Svevo TGTAGTGTTTCATTAGAATAGCAATCCATCATTTTTTTCATGTTGCTTTCAGGATGTGGC 3203

Sus2_2A_Ciccio TGTAGCGTTTCATTAGAATGGCAATCCATAATTTTTTTCCTGTTGCTTTCAGGATGTGGC 3087

Sus2_2A_Svevo TGTAGCGTTTCATTAGAATGGCAATCCATAATTTTTTTCCTGTTGCTTTCAGGATGTGGC 3087

***** ************* ********* ********* ********************

Sus2_2B_Ciccio ACACGAGATCGCCGGAGAGCTGCAGGCCACTCCTGACCTGATCATTGGAAACTACAGTGA 3263

Sus2_2B_Svevo ACACGAGATCGCCGGAGAGCTGCAGGCCACTCCTGACCTGATCATTGGAAACTACAGTGA 3263

Sus2_2A_Ciccio ACACGAGATCGCCGGAGAGCTGCAGGCCACTCCTGACCTGATCATTGGAAACTACAGTGA 3147

Sus2_2A_Svevo ACACGAGATCGCCGGAGAGCTGCAGGCCACTCCTGACCTGATCATTGGAAACTACAGTGA 3147

************************************************************

Sus2_2B_Ciccio TGGCAACCTAGTCGCGTGTTTGTTGGCTCACAAGTTGGGAGTTACTCATGTACGCATTCT 3323

Sus2_2B_Svevo TGGCAACCTAGTCGCGTGTTTGTTGGCTCACAAGTTGGGAGTTACTCATGTACGCATTCT 3323

Sus2_2A_Ciccio TGGCAACCTAGTCGCATGTTTGTTAGCTCACAAGTTGGGAGTTACCCATGTACGCATTCT 3207

Sus2_2A_Svevo TGGCAACCTAGTCGCATGTTTGTTAGCTCACAAGTTGGGAGTTACCCATGTACGCATTCT 3207

*************** ******** ******************** **************

Sus2_2B_Ciccio ATCCCCTTTTGCTCAATACAAGATGTATTCTGATCTCCGTAGTATCCTATGATATTTCTT 3383

Sus2_2B_Svevo ATCCCCTTTTGCTCAATACAAGATGTATTCTGATCTCCGTAGTATCCTATGATATTTCTT 3383

Sus2_2A_Ciccio A-CCCCTTTTGCTCAGTACAAGATGTATTCTCATCTCCGTAGTATCCTATCATAATTCTT 3266

Sus2_2A_Svevo A-CCCCTTTTGCTCAGTACAAGATGTATTCTCATCTCCGTAGTATCCTATCATAATTCTT 3266

* ************* *************** ****************** *** *****

Sus2_2B_Ciccio ATTTCCTCATGTGCAGTGTACCATTGCGCATGCACTCGAGAAAACCAAGTATCCCAACTC 3443

Sus2_2B_Svevo ATTTCCTCATGTGCAGTGTACCATTGCGCATGCACTCGAGAAAACCAAGTATCCCAACTC 3443

Sus2_2A_Ciccio ATTTCCTCATGTGCAGTGTACCATTGCTCATGCACTTGAGAAAACCAAGTATCCCAACTC 3326

Sus2_2A_Svevo ATTTCCTCATGTGCAGTGTACCATTGCTCATGCACTTGAGAAAACCAAGTATCCCAACTC 3326

*************************** ******** ***********************

Sus2_2B_Ciccio CGACCTTTACTGGAAGAAATTTGAGGATCACTACCACTTCTCCTGCCAGTTCACAGCTGA 3503

Sus2_2B_Svevo CGACCTTTACTGGAAGAAATTTGAGGATCACTACCACTTCTCCTGCCAGTTCACAGCTGA 3503

Sus2_2A_Ciccio CGACCTTTACTGGAAGAAATTCGAGGATCACTACCACTTCTCCTGCCAGTTTACAGCCGA 3386

Sus2_2A_Svevo CGACCTTTACTGGAAGAAATTCGAGGATCACTACCACTTCTCCTGCCAGTTTACAGCCGA 3386

********************* ***************************** ***** **

Sus2_2B_Ciccio CCTGATTGCAATGAATCATGCTGACTTCATCATCACCAGTACTTTCCAAGAGATTGCCGG 3563

Sus2_2B_Svevo CCTGATTGCAATGAATCATGCTGACTTCATCATCACCAGTACTTTCCAAGAGATTGCCGG 3563

Sus2_2A_Ciccio CCTGATTGCAATGAACCATGCCGACTTCATCATCACCAGTACTTTCCAAGAGATTGCCGG 3446

Sus2_2A_Svevo CCTGATTGCAATGAACCATGCCGACTTCATCATCACCAGTACTTTCCAAGAGATTGCCGG 3446

*************** ***** **************************************

Sus2_2B_Ciccio AAAGTAAGATTCTCTCTTTCACAGAAACACCGCCATGGTCATTTGCAAATAGCATAAACT 3623

Sus2_2B_Svevo AAAGTAAGATTCTCTCTTTCACAGAAACACCGCCATGGTCATTTGCAAATAGCATAAACT 3623

Sus2_2A_Ciccio AAAGTAAGATTCTCTCTTTCACAGAAACACCGCCGTGGTCATTTGCAAATAGGATAAACT 3506

Sus2_2A_Svevo AAAGTAAGATTCTCTCTTTCACAGAAACACCGCCGTGGTCATTTGCAAATAGGATAAACT 3506

********************************** ***************** *******

Sus2_2B_Ciccio GATCTAATGTT-ATCGTCCAATTTTCAGCAAGGACACCGTAGGGCAGTACGAGTCGCACA 3682

Sus2_2B_Svevo GATCTAATGTT-ATCGTCCAATTTTCAGCAAGGACACCGTAGGGCAGTACGAGTCGCACA 3682

Sus2_2A_Ciccio GATCTAATGTTTATCGTTCAATTTTCAGCAAGGACACCGTAGGGCAGTACGAGTCGCACA 3566

Sus2_2A_Svevo GATCTAATGTTTATCGTTCAATTTTCAGCAAGGACACCGTAGGGCAGTACGAGTCGCACA 3566

*********** ***** ******************************************

Sus2_2B_Ciccio TGGCATTCACAATGCCAGGCCTCTATCGTGTTGTCCATGGTATTGATGTCTTCGACCCCA 3742

Sus2_2B_Svevo TGGCATTCACAATGCCAGGCCTCTATCGTGTTGTCCATGGTATTGATGTCTTCGACCCCA 3742

Sus2_2A_Ciccio TGGCATTCACAATGCCTGGCCTCTATCGTGTTGTCCATGGTATTGATGTCTTCGACCCCA 3626

Sus2_2A_Svevo TGGCATTCACAATGCCTGGCCTCTATCGTGTTGTCCATGGTATTGATGTCTTCGACCCCA 3626

**************** *******************************************

Sus2_2B_Ciccio AGTTCAACATCGTCTCCCCTGGTGCTGACATGTCCATCTACTTCCCATACACTGAACAGC 3802

Sus2_2B_Svevo AGTTCAACATCGTCTCCCCTGGTGCTGACATGTCCATCTACTTCCCATACACTGAACAGC 3802

Sus2_2A_Ciccio AGTTCAACATCGTCTCCCCTGGTGCTGACATGTCCATCTACTTCCCATACACTGAACAGC 3686

Sus2_2A_Svevo AGTTCAACATCGTCTCCCCTGGTGCTGACATGTCCATCTACTTCCCATACACTGAACAGC 3686

************************************************************

Sus2_2B_Ciccio AGAAGAGGCTTACCTCCCTCCATACTGAGATTGAGGAGCTACTCTTCAGTGATGTTGAGA 3862

Sus2_2B_Svevo AGAAGAGGCTTACCTCCCTCCATACTGAGATTGAGGAGCTACTCTTCAGTGATGTTGAGA 3862

Sus2_2A_Ciccio AGAAGAGGCTTACCTCCCTCCATACTGAGATTGAGGAGCTACTCTTCAGTGATATTGAGA 3746

Sus2_2A_Svevo AGAAGAGGCTTACCTCCCTCCATACTGAGATTGAGGAGCTACTCTTCAGTGATATTGAGA 3746

***************************************************** ******

Sus2_2B_Ciccio ATGCTGAGCACAAGTATAATCATATATAAGTCTTGACTTCTTATAAAATAGATTGCCTAC 3922

Sus2_2B_Svevo ATGCTGAGCACAAGTATAATCATATATAAGTCTTGACTTCTTATAAAATAGATTGCCTAC 3922

Sus2_2A_Ciccio ATGCTGAGCACAAGTATAATCATATATAACTCTTGACTT---ATAAAATAGATTGCCCAC 3803

Sus2_2A_Svevo ATGCTGAGCACAAGTATAATCATATATAACTCTTGACTT---ATAAAATAGATTGCCCAC 3803

***************************** ********* *************** **

Sus2_2B_Ciccio CATGCTGGTTATCTAACTTGACTACCTCTTCAGATTTGTGCTGAAGGACAAGAAGAAGCC 3982

Sus2_2B_Svevo CATGCTGGTTATCTAACTTGACTACCTCTTCAGATTTGTGCTGAAGGACAAGAAGAAGCC 3982

Sus2_2A_Ciccio CGTGCTGGTTATCTAACTTGACTACCTCTCCAGATTTGTGCTGAAGGACAAGAAGAAGCC 3863

Sus2_2A_Svevo CGTGCTGGTTATCTAACTTGACTACCTCTCCAGATTTGTGCTGAAGGACAAGAAGAAGCC 3863

* *************************** ******************************

Sus2_2B_Ciccio GATCATCTTCTCGATGGCTAGGCTGGACCGTGTCAAGAATATGACTGGCCTGGTAGAAAT 4042

Sus2_2B_Svevo GATCATCTTCTCGATGGCTAGGCTGGACCGTGTCAAGAATATGACTGGCCTGGTAGAAAT 4042

Sus2_2A_Ciccio GATCATCTTCTCGATGGCTAGGTTGGACCGTGTCAAGAATATGACTGGCCTGGTAGAAAT 3923

Sus2_2A_Svevo GATCATCTTCTCGATGGCTAGGTTGGACCGTGTCAAGAATATGACTGGCCTGGTAGAAAT 3923

********************** *************************************

Sus2_2B_Ciccio GTATGGGCGGAATCCTCGCCTACAGGAGCTGGTAAACCTGGTGGTTGTTTGTGGTGACCA 4102

Sus2_2B_Svevo GTATGGGCGGAATCCTCGCCTACAGGAGCTGGTAAACCTGGTGGTTGTTTGTGGTGACCA 4102

Sus2_2A_Ciccio GTATGGGCGGAATCCTCGCCTACAGGAGCTGGTAAACCTAGTGGTTGTCTGTGGTGACCA 3983

Sus2_2A_Svevo GTATGGGCGGAATCCTCGCCTACAGGAGCTGGTAAACCTAGTGGTTGTCTGTGGTGACCA 3983

*************************************** ******** ***********

Sus2_2B_Ciccio TGGAAAGGTGTCCAAGGACAAGGAGGAGCAGGCAGAGTTCAAAAAGATGTTTGATCTTAT 4162

Sus2_2B_Svevo TGGAAAGGTGTCCAAGGACAAGGAGGAGCAGGCAGAGTTCAAAAAGATGTTTGATCTTAT 4162

Sus2_2A_Ciccio TGGAAAGGTGTCCAAGGACAAGGAGGAGCAGGCAGAGTTCAAGAAGATGTTTGATCTTAT 4043

Sus2_2A_Svevo TGGAAAGGTGTCCAAGGACAAGGAGGAGCAGGCAGAGTTCAAGAAGATGTTTGATCTTAT 4043

****************************************** *****************

Sus2_2B_Ciccio CGAACAGTACAACCTGATTGGTCACATCCGCTGGATCTCTGCTCAGATGAACCGTGTCCG 4222

Sus2_2B_Svevo CGAACAGTACAACCTGATTGGTCACATCCGCTGGATCTCTGCTCAGATGAACCGTGTCCG 4222

Sus2_2A_Ciccio TGAACAGTACAACCTGATTGGACACATCCGCTGGATCTCTGCTCAGATGAACCGTGTCCG 4103

Sus2_2A_Svevo TGAACAGTACAACCTGATTGGACACATCCGCTGGATCTCTGCTCAGATGAACCGTGTCCG 4103

******************** **************************************

Sus2_2B_Ciccio CAATGGTGAGCTCTACCGCTACATCTGCGACATGAAGGGAGCCTTTGTGCAGGTGAAGGA 4282

Sus2_2B_Svevo CAATGGTGAGCTCTACCGCTACATCTGCGACATGAAGGGAGCCTTTGTGCAGGTGAAGGA 4282

Sus2_2A_Ciccio CAATGGTGAGCTCTACCGCTACATCTGCGACATGAAGGGAGCCTTTGTGCAGGTGAAGGA 4163

Sus2_2A_Svevo CAATGGTGAGCTCTACCGCTACATCTGCGACATGAAGGGAGCCTTTGTGCAGGTGAAGGA 4163

************************************************************

Sus2_2B_Ciccio CACTGTCAACCTAGCAACTCACTATGACTGAATCACGACATTTACTAGTTCTGACAATCT 4342

Sus2_2B_Svevo CACTGTCAACCTAGCAACTCACTATGACTGAATCACGACATTTACTAGTTCTGACAATCT 4342

Sus2_2A_Ciccio CACTGTCAACCTAGGAACTCACTATGACTGAATCACGACATTTACTAGTTCTGACGCTCT 4223

Sus2_2A_Svevo CACTGTCAACCTAGGAACTCACTATGACTGAATCACGACATTTACTAGTTCTGACGCTCT 4223

************** **************************************** ***

Sus2_2B_Ciccio TTTTTCATGTTTGGCTTCCTGTGCTCAGCCTGCTTTCTATGAGGCTTTCGGTCTTACCGT 4402

Sus2_2B_Svevo TTTTTCATGTTTGGCTTCCTGTGCTCAGCCTGCTTTCTATGAGGCTTTCGGTCTTACCGT 4402

Sus2_2A_Ciccio TTTTTCATGTTTGGCTTCCTGTGCTCAGCCTGCTTTCTATGAGGCTTTCGGTCTTACTGT 4283

Sus2_2A_Svevo TTTTTCATGTTTGGCTTCCTGTGCTCAGCCTGCTTTCTATGAGGCTTTCGGTCTTACTGT 4283

********************************************************* **

Sus2_2B_Ciccio GATAGAGGCCATGACATGTGGCCTTCCAACATTCGCCACTGCATATGGTGGTCCAGCTGA 4462

Sus2_2B_Svevo GATAGAGGCCATGACATGTGGCCTTCCAACATTCGCCACTGCATATGGTGGTCCAGCTGA 4462

Sus2_2A_Ciccio GATAGAGGCCATGACATGTGGCCTTCCAACATTCGCCACTGCATATGGTGGTCCAGCTGA 4343

Sus2_2A_Svevo GATAGAGGCCATGACATGTGGCCTTCCAACATTCGCCACTGCATATGGTGGTCCAGCTGA 4343

************************************************************

Sus2_2B_Ciccio GATCATTGTGCACGGTGTGTCCGGCTACCACATCGATCCTTACCAGAATGACAAGGCCTC 4522

Sus2_2B_Svevo GATCATTGTGCACGGTGTGTCCGGCTACCACATCGATCCTTACCAGAATGACAAGGCCTC 4522

Sus2_2A_Ciccio GATCATTGTGAATGGTGTGTCCGGCTACCACATCGATCCTTACCAGAATGACAAGGCCTC 4403

Sus2_2A_Svevo GATCATTGTGAATGGTGTGTCCGGCTACCACATCGATCCTTACCAGAATGACAAGGCCTC 4403

********** * ***********************************************

Sus2_2B_Ciccio CGCACTGCTTGTGGACTTCTTTGGGAAGTGCCAGGAAGACCCGAGCCACTGGAACAAGAT 4582

Sus2_2B_Svevo CGCACTGCTTGTGGACTTCTTTGGGAAGTGCCAGGAAGACCCGAGCCACTGGAACAAGAT 4582

Sus2_2A_Ciccio CGCACTGCTTGTGGACTTCTTTGGGAAGTGCAAGGAAGACCCGAGCCACTGGAACAAGAT 4463

Sus2_2A_Svevo CGCACTGCTTGTGGACTTCTTTGGGAAGTGCAAGGAAGACCCGAGCCACTGGAACAAGAT 4463

******************************* ****************************

Sus2_2B_Ciccio CTCGCAGGGAGGACTCCAGCGCATCGAGGAGAAGTATATAAGCAATTCTCTATCGTCTAC 4642

Sus2_2B_Svevo CTCGCAGGGAGGACTCCAGCGCATCGAGGAGAAGTATATAAGCAATTCTCTATCGTCTAC 4642

Sus2_2A_Ciccio CTCGCAGGGAGGACTCCAGCGCATCGAGGAGAAGTATATATGCAATTCTCTGTTACCTAT 4523

Sus2_2A_Svevo CTCGCAGGGAGGACTCCAGCGCATCGAGGAGAAGTATATATGCAATTCTCTGTTACCTAT 4523

**************************************** ********** * ***

Sus2_2B_Ciccio ATGTGTTATCATCTGATGCACATGCTCTGACTCTTTTAACAGCACTGAGCTGAGATTGAT 4702

Sus2_2B_Svevo ATGTGTTATCATCTGATGCACATGCTCTGACTCTTTTAACAGCACTGAGCTGAGATTGAT 4702

Sus2_2A_Ciccio CTATGTTACCATCTGATGCACATGCTCTGACTCTTTTAACAGCACTGAGCTGAGATTGAT 4583

Sus2_2A_Svevo CTATGTTACCATCTGATGCACATGCTCTGACTCTTTTAACAGCACTGAGCTGAGATTGAT 4583

* ***** ***************************************************

Sus2_2B_Ciccio GCCCTGTGATCTTGACGCAGGTACACCTGGAAGCTGTACTCTGAGAGGCTGATGACCCTT 4762

Sus2_2B_Svevo GCCCTGTGATCTTGACGCAGGTACACCTGGAAGCTGTACTCTGAGAGGCTGATGACCCTT 4762

Sus2_2A_Ciccio GCCCTGGGATCTTGACGCAGGTACACCTGGAAGCTGTACTCTGAGAGGTTGATGACCCTT 4643

Sus2_2A_Svevo GCCCTGGGATCTTGACGCAGGTACACCTGGAAGCTGTACTCTGAGAGGTTGATGACCCTT 4643

****** ***************************************** ***********

Sus2_2B_Ciccio TCTGGTGTCTATGGTTTCTGGAAGTATGTCTCCAACCTCGACAGGCGCGAGACTCGTCGC 4822

Sus2_2B_Svevo TCTGGTGTCTATGGTTTCTGGAAGTATGTCTCCAACCTCGACAGGCGCGAGACTCGTCGC 4822

Sus2_2A_Ciccio TCTGGTGTCTATGGTTTCTGGAAGTACGTCTCCAACCTCGACAGGCGCGAGACTCGTCGC 4703

Sus2_2A_Svevo TCTGGTGTCTATGGTTTCTGGAAGTACGTCTCCAACCTCGACAGGCGCGAGACTCGTCGC 4703

************************** *********************************

Sus2_2B_Ciccio TACCTTGAAATGCTCTACGCCCTCAAGTACCGCAAAATGGTATGTGCCATGACATTGCGT 4882

Sus2_2B_Svevo TACCTTGAAATGCTCTACGCCCTCAAGTACCGCAAAATGGTATGTGCCATGACATTGCGT 4882

Sus2_2A_Ciccio TACCTTGAAATGCTCTACGCCCTCAAGTACCGCAAAATGGTATGTGCCATGACATTGCGT 4763

Sus2_2A_Svevo TACCTTGAAATGCTCTACGCCCTCAAGTACCGCAAAATGGTATGTGCCATGACATTGCGT 4763

************************************************************

Sus2_2B_Ciccio TCGACCACCTTGATAATTTCAGCTCGGTTCATATCTTGAGACTTAATTTCTCTGTGCCTT 4942

Sus2_2B_Svevo TCGACCACCTTGATAATTTCAGCTCGGTTCATATCTTGAGACTTAATTTCTCTGTGCCTT 4942

Sus2_2A_Ciccio TCGACCACCTTGATAATTTCAGCTCGGTTCATGTCTTGAGACTTAATTTCTCTGTGCCTT 4823

Sus2_2A_Svevo TCGACCACCTTGATAATTTCAGCTCGGTTCATGTCTTGAGACTTAATTTCTCTGTGCCTT 4823

******************************** ***************************

Sus2_2B_Ciccio TGTTGCGTGCAGGCTGCAACTGTCCCATTGGCTGTTGAGGGCGAGACCTCGGGCAAATGA 5002

Sus2_2B_Svevo TGTTGCGTGCAGGCTGCAACTGTCCCATTGGCTGTTGAGGGCGAGACCTCGGGCAAATGA 5002

Sus2_2A_Ciccio TGTTGTGTGCAGGCTGAAACTGTCCCATTGGCTGTTGAGGGCGAGACCTCGGGCAAATGA 4883

Sus2_2A_Svevo TGTTGTGTGCAGGCTGAAACTGTCCCATTGGCTGTTGAGGGCGAGACCTCGGGCAAATGA 4883

***** ********** *******************************************

Sus2_2B_Ciccio TTTGTCCTTACCAGAGAAATAAATGGCGGGCGAGCGCTCCGCTTTACCGTTCTTGATTCA 5062

Sus2_2B_Svevo TTTGTCCTTACCAGAGAAATAAATGGCGGGCGAGCGCTCCGCTTTACCGTTCTTGATTCA 5062

Sus2_2A_Ciccio TTTGTCCTTACCAGAGAAATAAATGGCGGGCGAGCGCTCCGCTTTACCGTTCTTGATTCA 4943

Sus2_2A_Svevo TTTGTCCTTACCAGAGAAATAAATGGCGGGCGAGCGCTCCGCTTTACCGTTCTTGATTCA 4943

************************************************************

Sus2_2B_Ciccio GTGATGAAGCACAGATCGGAGAGTGTTATGCCTTTGATTGTCCTTTGTTACCGTTCTTGA 5122

Sus2_2B_Svevo GTGATGAAGCACAGATCGGAGAGTGTTATGCCTTTGATTGTCCTTTGTTACCGTTCTTGA 5122

Sus2_2A_Ciccio GTGATGGAGCACAGATCGGAGGGTGTTATGCCTTTGCTTGTCCTTTGTTACCGTTCTTGA 5003

Sus2_2A_Svevo GTGATGGAGCACAGATCGGAGGGTGTTATGCCTTTGCTTGTCCTTTGTTACCGTTCTTGA 5003

****** ************** ************** ***********************

Sus2_2B_Ciccio TTCAGT-ATGAACTTTAGTTCCTGTTGAGGCCCCGGCCGTGTTGTCGCGGTAGGGGAACT 5181

Sus2_2B_Svevo TTCAGT-ATGAACTTTAGTTCCTGTTGAGGCCCCGGCCGTGTTGTCGCGGTAGGGGAACT 5181

Sus2_2A_Ciccio TTCAGTGATGAACTTT--TTCCTGTTGAGTCCCCG----TGTTGTCGCGGTAGGGGAACT 5057

Sus2_2A_Svevo TTCAGTGATGAACTTT--TTCCTGTTGAGTCCCCG----TGTTGTCGCGGTAGGGGAACT 5057

****** ********* *********** ***** *********************

Sus2_2B_Ciccio GGATGGATGCTGTGTTGTTGGTACTTTCTCATGGATACAGTATTTGAATGAATGAATAAG 5241

Sus2_2B_Svevo GGATGGATGCTGTGTTGTTGGTACTTTCTCATGGATACAGTATTTGAATGAATGAATAAG 5241

Sus2_2A_Ciccio GGATGGATGCTGTGTTGTTGGTACTTTCTCATGGATACAGTATTTGAATGAATGAATAAG 5117

Sus2_2A_Svevo GGATGGATGCTGTGTTGTTGGTACTTTCTCATGGATACAGTATTTGAATGAATGAATAAG 5117

************************************************************

Sus2_2B_Ciccio ATAGTCTTTGTTTT 5255

Sus2_2B_Svevo ATAGTCTTTGTTT- 5254

Sus2_2A_Ciccio ATAGTCTTTGTTTT 5131

Sus2_2A_Svevo ATAGTCTTTGTTTT 5131

**Figure S2.** **Primers location in the two *Sus2-2A* and *Sus2-2B* genomic sequences.**

Boxed in yellow the initial codon ATG and the stop codon TGA; exons are delimitated by red brackets; primers are indicated by an arrow

Sus2_2B CGGAGTTTCTGGAATGCAAAAGTAAATCTAGCAGGTTTTTTT--CTTCCTATTTTATGCA 58

FOR-5’prom

Sus2_2A CGGAGTTTCTGGAATGCAAAAGTAAATCTAGCAGTTTTTTTTTGCTTCCTATTTTACGCA 60

Sus2_2B ACTTGTTAAAGGGGCTATTAAAGTTTT------ATCAAGGCCAGTAATTAAGAGTTACAG 112

Sus2_2A ACTTGTTAAA---GCTATTAACTTTTCCTGCAGAGCAAAGCTATTAACTCTGTACTCCAA 117

Sus2_2B TGGTTCTC----ACTAATC-CTACATAAGCTTCTTGCCTTGCGGATAATGTGCATGTTTT 167

Sus2_2A AGTTTTTCCAAGGCCAGTAATTAATTAAGCTTCTTGCCTTGTGGATCATGTGCATGTCAT 177

Sus2_2B TGTATTACTACTGTACTAAATTAGCTGATCCGTTTCTATTTATTTGCATGCTCTCTACTG 227

Sus2_2A TGTATAA------TACTA------------------------------------------ 189

Sus2_2B GAAAAGTGGAGAGCCTGCCCGGCAATAGTCCCGTGCTCCTGTGCTCACTCATATTTTGGT 287

Sus2_2A -------GGAGTAC---------------------------------------------- 196

Sus2_2B CAGTGTTAATTACTTGATCTGTTTCTAGTATCTGCATGCCCTTTACTGGAAAACTGGAGA 347

Sus2_2A -----TAAATTACTTGATCCGTTTCTATTATCTGCATGTCCTCCATTGGAAAACTGGAGA 251

Sus2_2B GCCTGCCCGGCAATAGTTCTGTCTTCTGTGCTCCTGCGCTCACTCATATACTTTGGTCAG 407

Sus2_2A GCCTGCCCGGCAATAGTTCCGT-------GCTCCTGTGCTCACTCATATACTTTGGTCAG 304

Sus2_2B TTTTAATTAGCTTGCCAAGCCACGCAAGGAACGACTAGGATACATCTCACGCTAATCCCA 467

Sus2_2A TTTTAATTAGCTTGCCAAGCCACGCAAGAAACGGCTACAATACATCTCACGCTAATCCCA 364

Sus2_2B TGATCTGCTTCCCAAAGCACAACCTTCTAGGTACTCTTGCACGTCACGCCAACCTTTCTT 527

Sus2_2A TGATCTGGTTCCCAAAGCACAGGCTTCTAGGCACTCTTGCACGCCACGCCAACCTTTCTT 424

Sus2_2B CTTCCTTTGGCTCTATTTATCAGTAGAGCTCATGCATCGTTGCTACTAGGACAGAGCGCC 587

Sus2_2A CTTCCTCCGGCTCTATATATCAGTAGAGCTCATGCATCGTTGCC---AGGACAGAGCGCC 481

Sus2_2B CTGTTGTCTAGGAGGAGGCTTCACTGTTTCTGCTGTTCAGGTTTACATGTCTGTTTCCGC 647

Sus2_2A CTGTTGTGTGGGAGGAGGCTCCACTGTTTCTGCTGTTCAGATTTACATGACTGTTTCCGC 541

Sus2167-for

Sus2_2B TGTTCAGGTTTGAGGACAGCAATGGGGGAGACTGCCGGAGAGCGCGCCCTGAGCCGCATC 707

Sus2_2A TGTTCAGGTTTGAGGACAGCAATGGGGGAGACTGCCGGAGAGCGCGCCCTGAGCCGCATC 601

Sus2_2B CACAGCGTGAGGGAGCGCATCGGCGATTCCCTCTCCGCGCACACCAATGAGCTTGTCGCC 767

Sus2_2A CACAGCGTGAGGGAGCGCATCGGTGATTCCCTCTCTGCGCACACCAATGAGCTCGTCGCC 661

SUS2-REV9

Sus2_2B GTCTTCTCAAGGTCTGTCCTTGATCTTTTGCAGGCTAAACTGGCTGCCGGTTTGTAGAAA 827

Sus2_2A GTCTTCTCAAGGTCTGTCCCTGATCTTTTGCAGGCTAGACTGGCTGCCAATTTGTACAAA 721

Sus2_2B TCTCTGCATATAAGAATGATATGGTTAAGCGATGAATTATGGCTGCCTTGTGTATTTCTA 887

Sus2_2A TCTCTGCATGTGAG----ACACGGTTAAGCGATGAATTATGGTTGCCTTGCGTATTTCTA 777

Sus2_2B TGTTATGCTATAGTATGAGCTGGATCAATGGCTTTTTTCTTTAAAAAGTCTTGTTACTAA 947

Sus2_2A TGTCATGCTATAGTATGAGCTGGATCAATGATTTTTTTCTTTACAAAAT-TTGT-ACCAA 835

SUS2-REV7

Sus2_2B GGACCATTAAAAGAACTGATGATGAAGTTTCAACAATTGTTCCTGCATTTCTATATAGTA 1007

Sus2_2A GGGCCATTAAAAGAACTGATGATGAAGTTTCAACAATTGTTCCTGCATTTCTATATAATA 895

Sus2_2B CTGCTCCTTCTATTTGTCTTTTAACTTCTTTCTTAAT-CTAGTTGTATTTATACTATAAC 1066

Sus2_2A CCGCTCCTTCCATTTGTCTTTTAACTTCTTTCTTAATACTAGTTGTATTCATACTATAAC 955

Sus2_2B AAAGTACCTGAACATTTTCTTACTGCTACAATTGTTACCATACTTTGTTCTTCAAGTAAC 1126

Sus2_2A AAAGTACCTGAACATTTTCTTACTGCTACAATTGTTACCACACTTTGT---TCAAGTAAC 1012

Sus2_2B GTTTGTTGATAGATCTGACCCACTGGCTGTTACATGATAACTTGTTCAGGCTTGTTAACC 1186

Sus2_2A GTTTGTTGATAGATCTGACCCACCTGCTGTTACACGATAACTTGTTCAGGCTTGTTAACC 1072

FOR-ESII

Sus2_2B AAGGAAAGGGGATGCTGCAGCCCCATCAGATAACTGCTGAGTACAATGCCGCGATCCCAG 1246

Sus2_2A AAGGAAAGGGGATGCTGCAGCCCCATCAGATCACTGCTGAGTACAATGCCGCGATCCCTG 1132

Rev1-ciccio

Sus2_2B AGGCCGAGCGTGAGAAGCTCAAGGACACCGCCTTTGAGGATCTCCTAAGGGGCGCACAGG 1306

Sus2_2A AGGCCGAGCGCGAGAAGCTCAAGGACACCGCCTTTGAGGATCTCCTAAGGGGCGCACAGG 1192

Sus2_2B TTTGCACCACCAAAACTCACTGCAGTGTCATTCAAGTGATGTTCAGCTCTTGATCTGTTG 1366

Sus2_2A TTTGCACCACCAAAACGCACTGCACTGTCATTCCAGTGATGTTCAGCTCTTGATCTGTTG 1252

Sus2_2B GTTTCTCATACAGGAGGCAATTGTCATCCCTCCATGGGTTGCTCTCGCCATCCGGCCAAG 1426

Sus2_2A GTTTCTCATATAGGAGGCAATTGTCATCCCTCCATGGGTTGCTCTCGCCATCCGGCCAAG 1312

Sus2_2B GCCCGGCGTCTGGGAGTATGTGAGGGTCAATGTGAGCGAGCTTGGTGTTGAGGAGTTAAG 1486

Sus2_2A GCCCGGCGTCTGGGAGTATGTGAGGGTCAATGTGAGCGAGCTTGGTGTTGAGGAGTTGAG 1372

Sus2_2B CATCGCTGAGTATTTGCAGTTCAAGGAACAACTGGCGAATGGAAGGTATCTGTGATTTTG 1546

Sus2_2A CGTCGCTGAGTATTTGCAGTTCAAGGAACAACTGGCGAATGGAAGGTATCTGTGGTTTTG 1432

Sus2_2B TGAAATCATTAAGATTCAAGAGTCCACCTTATACCTTAGTTTT-ATCATACAACGCTTCT 1605

Sus2_2A TGAAATTATTAAGATTCAAGAAGTCAGATTATACCTTAGTTTTTATTATATAACACTTCT 1492

Sus2_2B TCTGTTCAAATTGCAGCATCGATAACAACTTTGTGCTTGAGCTGGACTTTGAGCCATTCA 1665

Sus2_2A TCTGTTCAAATTGCAGCATCGATAACAACTTTGTGCTTGAGCTGGACTTTGAGCCATTCA 1552

Sus2_2B ACGCCTCCTTCCCTCGCCCATCGCTGTCGAAGTCCATTGGCAACGGTGTGCAGTTTCTGA 1725

Sus2_2A ACGCCTCCTTCCCGCGCCCATCGCTGTCGAAGTCCATTGGCAACGGTGTGCAGTTCCTGA 1612

Sus2_2B ACAGGCACTTGTCATCGAAGCTGTTCCATGACAAGGAGAGCATGTACCCATTGCTCAACT 1785

Sus2_2A ACAGGCACTTGTCATCGAAGCTGTTCCATGACAAGGAGAGCATGTACCCATTGCTCAACT 1672

Sus2_2B TCCTTCGCGCGCACAACTACAAGGGGATGGTAGGTTACACTCTCCAGTGTCTGGCTCTGT 1845

Sus2_2A TCCTTCGCGCGCACAACTACAAGGGGATGGTAGGTTACACTCTCCAGTGTCTTGCTTTGT 1732

Sus2_2B AGATTGGATCATTTGGCTTTGTAGATTGGATCATTCAACTATATCTTTGCTGAAGGTTCT 1905

Sus2_2A AGATTGGATCGTTTGGCTTTGTAGATTGGATGATTCGACTATATCTTTGCTGAAGGTTCT 1792

Sus2_2B CTAGAAAAACTATATCTTTGTTGTTGAA--------GGCTTACATCATTTGTTTATAGCA 1957

Sus2_2A ATAGAAA--CTACATCTTTGTTGTTGAAAACTGACAGGCTTACATCATTTGTTAATGGCA 1850

Sus2_2B TGTCAATCTCTTAGATATATCTAAAAGCAGCATATGAGCATACCCAGCCTGTAAGAAAAT 2017

Sus2_2A TGGCGATCTCTTAGATATATCTAAAGGCAGCATATGAGCATAGTCAGCCTGCAAGAAAAT 1910

Sus2_2B ATAGATACAATCT-ACTTAATGTGTTGGGCATTTGCCCTGATGATATGTGCATTT-GTAA 2075

Sus2_2A ATAGACACGATCTTACTTAATGTGTCAGGCATTTGCCCTGATGATATGTGCATTTTGTAT 1970

Sus2_2B GTAAAAAAAAAGTTTTAGAACAAATTCTTTAAG-AAGTGCAAAAGCCTGATTGCTATCTA 2134

Sus2_2A GTAAAAAAA--GTTTTAGAACAAATTCTTTAAGTAAGTGCAAAAGCCTGATTGCTATCTA 2028

Sus2_2B TGAGCCAACTGAAAGTTTAAACTGGAACTATTAGACCTGTTGTTTAATCACAATGAGCAT 2194

Sus2_2A TGAGCCAACTGAAAGTTTAAACTGGAACTACCATACCTGCTGCTTAATCATGACCAGCAT 2088

Sus2_2B GTAATATTTTCTTTCTTTCTTCAGACCATGATGTTGAACGACAGAATTCGGAGTCTCAGT 2254

Sus2_2A GTAATATTTTCTTTCTTTCTTCAGACCATGATGTTGAACGACAGGATTCGCAGTCTCGGT 2148

Sus2_2B ACCCTCCAAGGTGCACTCAGGAAGGCAGAGACACATCTGTCAGGCCTTCCAGCTGACACC 2314

Sus2_2A ACCCTCCAAGGTGCACTCAGGAAGGCAGAGACACATCTGTCAGGCCTTCCAGCTGACACC 2208

Sus2_2B CCTTACTCGGAGTTCCACCACCGGTACTGTATATAATCATCATACACATGATCATTGAAA 2374

Sus2_2A CCTTACTCAGAGTTCCACCACCGGTACTGTATATAATCATCATACACATGATCACTGAAA 2268

Sus2_2B CCCTTATTGCTCTCAACAAAGAACTAAAATCGTTGCGTATCAATTTTTTCGTTGCATAAT 2434

Sus2_2A TCCTTATTGTTCTCAACAAAGAACTAAAATCGTTGC-------------------ATAAT 2309

REV-ESVI

Sus2_2B TCAGGTTCCAGGAACTTGGTCTGGAGAAAGGTTGGGGCGACTGTGCTCAGCGTGCGAGTG 2494

Sus2_2A TCAGGTTCCAGGAACTTGGTTTGGAGAAAGGTTGGGGCGACTGTGCTCAGCGTGCGAGCG 2369

Sus2_2B AGACTATCCACCTTCTCTTGGACCTTCTCGAGGCCCCTGATCCATCCTCCTTGGAGAAGT 2554

Sus2_2A AGACTATCCACCTTCTCTTGGACCTTCTCGAGGCCCCTGATCCATCCTCCTTGGAGAAGT 2429

RT-6E-For2

Sus2_2B TCCTCGGAACAATCCCAATGGTGTTCAATGTTGTTATCCTCTCTCCTCATGGTTACTTTG 2614

Sus2_2A TCCTCGGGACAATCCCAATGGTGTTCAATGTCGTTATCCTCTCTCCTCATGGTTACTTTG 2489

RT-6E-For1

FOR-ESVI

Sus2_2B CTCAGGCCAATGTCTTGGGGTACCCTGATACTGGTGGACAGGTAGAATCCTCTACCCCAC 2674

Sus2_2A CTCAGGCCAATGTCTTGGGGTACCCTGATACTGGTGGACAGGTAGAATCCCCTACATCAT 2549

Sus2_2B TTTTGACAGCTTGACATATTTCCTCTTGATAAACTGAACACCAGGAAATATTTATTATCA 2734

Sus2_2A TTTTGACAGTTTGACATATTTCCTCTTGATAAAATGAACACTATGAAATCTTTGTTATCA 2609

Sus2_2B CCAATTTTACTATGTCATGGCA---CACAGATTGTCTACATTTTGGACCAAGTCCGTGCT 2791

Sus2_2A CCAATTATCTTATGTCATGGCAACCCATAGGTTGTTTACATTTTGGACCAAGTCCGTGCT 2669

RT-7E-Rev2

Sus2_2B ATGGAGAATGAGATGCTGTTGAGAATCAAGCAGCAAGGTCTTGACATTACACCAAAGATT 2851

Sus2_2A ATGGAGAATGAGATGCTGTTGAGAATCAAGCAGCAAGGTCTCGACATTACACCAAAGATT 2729

RT-7E-Rev1

Sus2_2B CTAATAGTAAGTTTAGTACCCCCAATATGATCGAATATGAACTCTCTATTTTAA------ 2905

Sus2_2A CTAATTGTAAGTTTAGTACC--CAACATGATCGAATATGAGCTCTCTGTTATAAACTTAT 2787

Sus2_2B --TTGATCCCTAATGAGGTATCTTTGTCCTGACATAGGTCACCAGGTTGCTCCCTGATGC 2963

Sus2_2A AATTGATTCCTAATGAGGTCTCTTTGGCCTTACATAGGTCACCAGGTTGCTCCCTGATGC 2847

Sus2_2B ACATGGCACCACCTGTGGCCAGCGCCTTGAGAAGGTCCTTGGCACCGAGCACACCCACAT 3023

Sus2_2A ACATGGCACCACCTGTGGCCAGCGCCTCGAGAAGGTCCTTGGCACCGAGCACACCCACAT 2907

Sus2_2B CCTGCGTGTGCCATTCAAAACAGAAGATGGTATTGTTCGCAAATGGATCTCCCGCTTTGA 3083

Sus2_2A CCTGCGTGTGCCATTCAAAACAGAAGATGGTATTGTTCGCAAATGGATCTCGCGTTTTGA 2967

Sus2_2B AGTCTGGCCTTACCTGGAAGCTTACACTGATGTATGCATTTCACTTTCCAGCAGGTTGTT 3143

Sus2_2A AGTCTGGCCTTACCTGGAAGCTTACACTGATGTATGCATTTCACTTTCCAGCAGGTTGTT 3027

Sus2_2B TGTAGTGTTTCATTAGAATAGCAATCCATCATTTTTTTCATGTTGCTTTCAGGATGTGGC 3203

Sus2_2A TGTAGCGTTTCATTAGAATGGCAATCCATAATTTTTTTCCTGTTGCTTTCAGGATGTGGC 3087

REV-ESIX

Sus2_2B ACACGAGATCGCCGGAGAGCTGCAGGCCACTCCTGACCTGATCATTGGAAACTACAGTGA 3263

Sus2_2A ACACGAGATCGCCGGAGAGCTGCAGGCCACTCCTGACCTGATCATTGGAAACTACAGTGA 3147

RT-9E-FORB

Sus2_2B TGGCAACCTAGTCGCGTGTTTGTTGGCTCACAAGTTGGGAGTTACTCATGTACGCATTCT 3323

Sus2_2A TGGCAACCTAGTCGCATGTTTGTTAGCTCACAAGTTGGGAGTTACCCATGTACGCATTCT 3207

RT-9E-FORA

Sus2_2B ATCCCCTTTTGCTCAATACAAGATGTATTCTGATCTCCGTAGTATCCTATGATATTTCTT 3383

Sus2_2A A-CCCCTTTTGCTCAGTACAAGATGTATTCTCATCTCCGTAGTATCCTATCATAATTCTT 3266

Sus2_2B ATTTCCTCATGTGCAGTGTACCATTGCGCATGCACTCGAGAAAACCAAGTATCCCAACTC 3443

Sus2_2A ATTTCCTCATGTGCAGTGTACCATTGCTCATGCACTTGAGAAAACCAAGTATCCCAACTC 3326

RT-10E-REVB

Sus2_2B CGACCTTTACTGGAAGAAATTTGAGGATCACTACCACTTCTCCTGCCAGTTCACAGCTGA 3503

Sus2_2A CGACCTTTACTGGAAGAAATTCGAGGATCACTACCACTTCTCCTGCCAGTTTACAGCCGA 3386

Sus2_2B CCTGATTGCAATGAATCATGCTGACTTCATCATCACCAGTACTTTCCAAGAGATTGCCGG 3563

Sus2_2A CCTGATTGCAATGAACCATGCCGACTTCATCATCACCAGTACTTTCCAAGAGATTGCCGG 3446

RT-10E-REVA

FOR-ESX

Sus2_2B AAAGTAAGATTCTCTCTTTCACAGAAACACCGCCATGGTCATTTGCAAATAGCATAAACT 3623

Sus2_2A AAAGTAAGATTCTCTCTTTCACAGAAACACCGCCGTGGTCATTTGCAAATAGGATAAACT 3506

Sus2_2B GATCTAATGTT-ATCGTCCAATTTTCAGCAAGGACACCGTAGGGCAGTACGAGTCGCACA 3682

Sus2_2A GATCTAATGTTTATCGTTCAATTTTCAGCAAGGACACCGTAGGGCAGTACGAGTCGCACA 3566

Sus2_2B TGGCATTCACAATGCCAGGCCTCTATCGTGTTGTCCATGGTATTGATGTCTTCGACCCCA 3742

Sus2_2A TGGCATTCACAATGCCTGGCCTCTATCGTGTTGTCCATGGTATTGATGTCTTCGACCCCA 3626

Sus2_2B AGTTCAACATCGTCTCCCCTGGTGCTGACATGTCCATCTACTTCCCATACACTGAACAGC 3802

Sus2_2A AGTTCAACATCGTCTCCCCTGGTGCTGACATGTCCATCTACTTCCCATACACTGAACAGC 3686

Sus2_2B AGAAGAGGCTTACCTCCCTCCATACTGAGATTGAGGAGCTACTCTTCAGTGATGTTGAGA 3862

Sus2_2A AGAAGAGGCTTACCTCCCTCCATACTGAGATTGAGGAGCTACTCTTCAGTGATATTGAGA 3746

Sus2_2B ATGCTGAGCACAAGTATAATCATATATAAGTCTTGACTTCTTATAAAATAGATTGCCTAC 3922

Sus2_2A ATGCTGAGCACAAGTATAATCATATATAACTCTTGACTT---ATAAAATAGATTGCCCAC 3803

Sus2_2B CATGCTGGTTATCTAACTTGACTACCTCTTCAGATTTGTGCTGAAGGACAAGAAGAAGCC 3982

Sus2_2A CGTGCTGGTTATCTAACTTGACTACCTCTCCAGATTTGTGCTGAAGGACAAGAAGAAGCC 3863

REV-ESXII

Sus2_2B GATCATCTTCTCGATGGCTAGGCTGGACCGTGTCAAGAATATGACTGGCCTGGTAGAAAT 4042

Sus2_2A GATCATCTTCTCGATGGCTAGGTTGGACCGTGTCAAGAATATGACTGGCCTGGTAGAAAT 3923

Sus2_2B GTATGGGCGGAATCCTCGCCTACAGGAGCTGGTAAACCTGGTGGTTGTTTGTGGTGACCA 4102

Sus2_2A GTATGGGCGGAATCCTCGCCTACAGGAGCTGGTAAACCTAGTGGTTGTCTGTGGTGACCA 3983

Sus2_2B TGGAAAGGTGTCCAAGGACAAGGAGGAGCAGGCAGAGTTCAAAAAGATGTTTGATCTTAT 4162

Sus2_2A TGGAAAGGTGTCCAAGGACAAGGAGGAGCAGGCAGAGTTCAAGAAGATGTTTGATCTTAT 4043

Sus2_2B CGAACAGTACAACCTGATTGGTCACATCCGCTGGATCTCTGCTCAGATGAACCGTGTCCG 4222

Sus2_2A TGAACAGTACAACCTGATTGGACACATCCGCTGGATCTCTGCTCAGATGAACCGTGTCCG 4103

FOR-ESXII

Sus2_2B CAATGGTGAGCTCTACCGCTACATCTGCGACATGAAGGGAGCCTTTGTGCAGGTGAAGGA 4282

Sus2_2A CAATGGTGAGCTCTACCGCTACATCTGCGACATGAAGGGAGCCTTTGTGCAGGTGAAGGA 4163

Sus2_2B CACTGTCAACCTAGCAACTCACTATGACTGAATCACGACATTTACTAGTTCTGACAATCT 4342

Sus2_2A CACTGTCAACCTAGGAACTCACTATGACTGAATCACGACATTTACTAGTTCTGACGCTCT 4223

Sus2_2B TTTTTCATGTTTGGCTTCCTGTGCTCAGCCTGCTTTCTATGAGGCTTTCGGTCTTACCGT 4402

Sus2_2A TTTTTCATGTTTGGCTTCCTGTGCTCAGCCTGCTTTCTATGAGGCTTTCGGTCTTACTGT 4283

Sus2_2B GATAGAGGCCATGACATGTGGCCTTCCAACATTCGCCACTGCATATGGTGGTCCAGCTGA 4462

Sus2_2A GATAGAGGCCATGACATGTGGCCTTCCAACATTCGCCACTGCATATGGTGGTCCAGCTGA 4343

FORB-3RACE

Sus2_2B GATCATTGTGCACGGTGTGTCCGGCTACCACATCGATCCTTACCAGAATGACAAGGCCTC 4522

Sus2_2A GATCATTGTGAATGGTGTGTCCGGCTACCACATCGATCCTTACCAGAATGACAAGGCCTC 4403

FORA-3RACE

FOR-ESXIII

Sus2_2B CGCACTGCTTGTGGACTTCTTTGGGAAGTGCCAGGAAGACCCGAGCCACTGGAACAAGAT 4582

Sus2_2A CGCACTGCTTGTGGACTTCTTTGGGAAGTGCAAGGAAGACCCGAGCCACTGGAACAAGAT 4463

REV-ESXIII

Sus2_2B CTCGCAGGGAGGACTCCAGCGCATCGAGGAGAAGTATATAAGCAATTCTCTATCGTCTAC 4642

Sus2_2A CTCGCAGGGAGGACTCCAGCGCATCGAGGAGAAGTATATATGCAATTCTCTGTTACCTAT 4523

Sus2_2B ATGTGTTATCATCTGATGCACATGCTCTGACTCTTTTAACAGCACTGAGCTGAGATTGAT 4702

Sus2_2A CTATGTTACCATCTGATGCACATGCTCTGACTCTTTTAACAGCACTGAGCTGAGATTGAT 4583

Sus2_2B GCCCTGTGATCTTGACGCAGGTACACCTGGAAGCTGTACTCTGAGAGGCTGATGACCCTT 4762

Sus2_2A GCCCTGGGATCTTGACGCAGGTACACCTGGAAGCTGTACTCTGAGAGGTTGATGACCCTT 4643

FORB2-3RACE

Sus2_2B TCTGGTGTCTATGGTTTCTGGAAGTATGTCTCCAACCTCGACAGGCGCGAGACTCGTCGC 4822

Sus2_2A TCTGGTGTCTATGGTTTCTGGAAGTACGTCTCCAACCTCGACAGGCGCGAGACTCGTCGC 4703

FORA2-3RACE

Sus2_2B TACCTTGAAATGCTCTACGCCCTCAAGTACCGCAAAATGGTATGTGCCATGACATTGCGT 4882

Sus2_2A TACCTTGAAATGCTCTACGCCCTCAAGTACCGCAAAATGGTATGTGCCATGACATTGCGT 4763

Sus2_2B TCGACCACCTTGATAATTTCAGCTCGGTTCATATCTTGAGACTTAATTTCTCTGTGCCTT 4942

Sus2_2A TCGACCACCTTGATAATTTCAGCTCGGTTCATGTCTTGAGACTTAATTTCTCTGTGCCTT 4823

REV-ESXV

Sus2_2B TGTTGCGTGCAGGCTGCAACTGTCCCATTGGCTGTTGAGGGCGAGACCTCGGGCAAATGA 5002

Sus2_2A TGTTGTGTGCAGGCTGAAACTGTCCCATTGGCTGTTGAGGGCGAGACCTCGGGCAAATGA 4883

Sus2_2B TTTGTCCTTACCAGAGAAATAAATGGCGGGCGAGCGCTCCGCTTTACCGTTCTTGATTCA 5062

Sus2_2A TTTGTCCTTACCAGAGAAATAAATGGCGGGCGAGCGCTCCGCTTTACCGTTCTTGATTCA 4943

Sus2168-rev

Sus2_2B GTGATGAAGCACAGATCGGAGAGTGTTATGCCTTTGATTGTCCTTTGTTACCGTTCTTGA 5122

Sus2_2A GTGATGGAGCACAGATCGGAGGGTGTTATGCCTTTGCTTGTCCTTTGTTACCGTTCTTGA 5003

Sus2_2B TTCAGT-ATGAACTTTAGTTCCTGTTGAGGCCCCGGCCGTGTTGTCGCGGTAGGGGAACT 5181

Sus2_2A TTCAGTGATGAACTTT--TTCCTGTTGAGTCCCCG----TGTTGTCGCGGTAGGGGAACT 5057

Sus2_2B GGATGGATGCTGTGTTGTTGGTACTTTCTCATGGATACAGTATTTGAATGAATGAATAAG 5241

Sus2_2A GGATGGATGCTGTGTTGTTGGTACTTTCTCATGGATACAGTATTTGAATGAATGAATAAG 5117

Sus2_2B ATAGTCTTTGTTTT 5255

Sus2_2A ATAGTCTTTGTTTT 5131

**Figure S3: Physical mapping of the *Sus2* genes.**

PCR amplification on nulli-tetrasomic lines N2A-T2B and N2B-T2A (lanes 1 and 3, respectively). Lanes 2 and 4 shows, respectively, the restriction digestions of PCR fragment of N2A-T2B (lane 1) and N2B-T2A (lane 3); Marker 1kb DNA Plus Ladder (M).


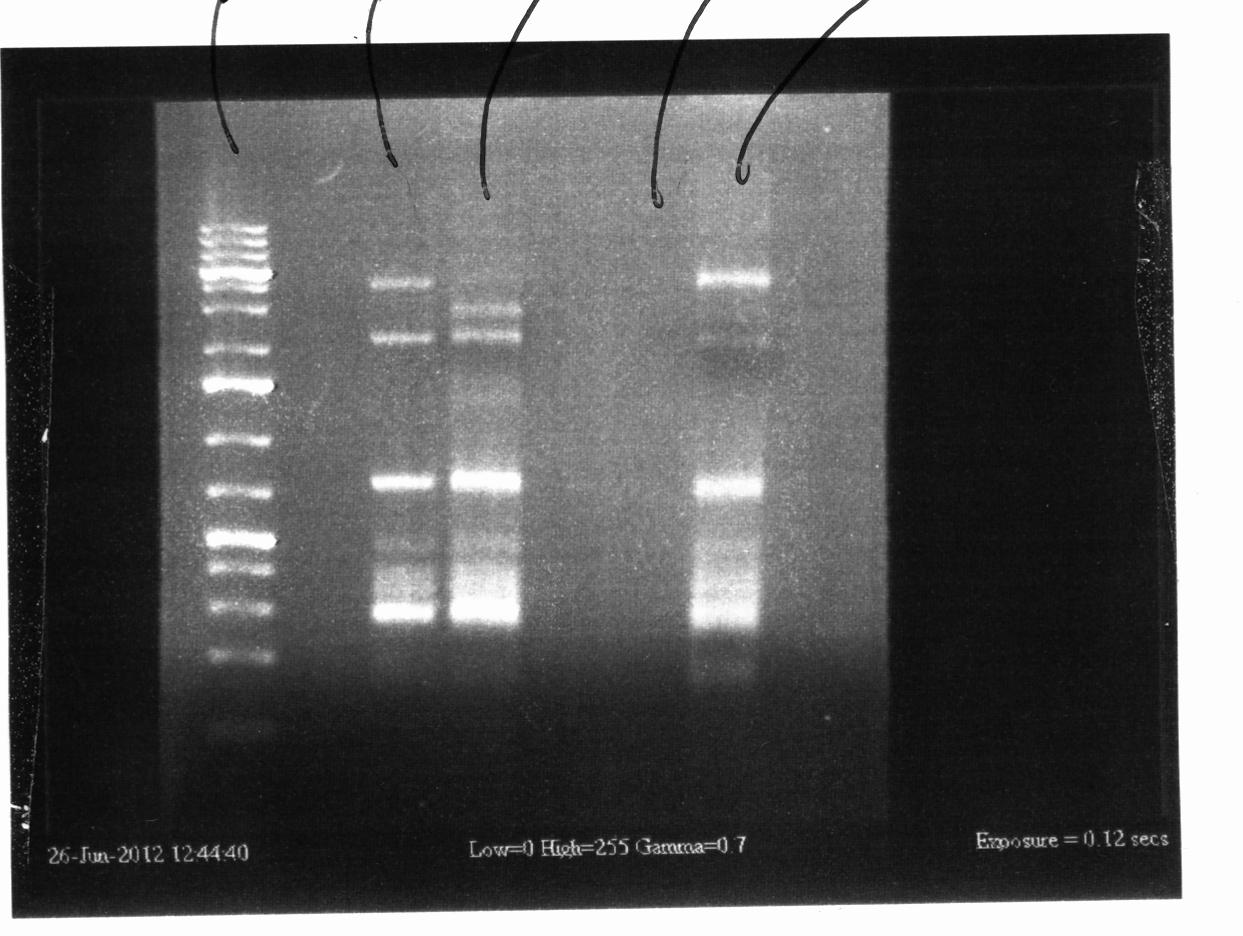

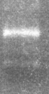


**M**

**1**

**2**

**3**

**4**

5000 bp

1500 bp
